# Avian interferon regulatory factor (IRF) family reunion: IRF3 and IRF9 found

**DOI:** 10.1186/s12915-025-02261-4

**Published:** 2025-07-01

**Authors:** Lenka Ungrová, Josef Geryk, Marina Kohn, Dana Kučerová, Veronika Krchlíková, Tomáš Hron, Vladimír Pečenka, Petr Pajer, Eliška Gáliková, Ľubomíra Pecnová, Bernd Kaspers, Jiří Hejnar, Jiří Nehyba, Daniel Elleder

**Affiliations:** 1 Laboratory of Viral and Cellular Genetics, Institute of Molecular Genetics of the Czech Academy of Sciences, Vídeňská 1083, Prague 4, 14220 Czech Republic; 2 Department for Veterinary Sciences, Ludwig-Maximilians-Universität Munich, 82152 Planegg, Germany; 3 Present Address: Centre International de Recherche en Infectiologie (CIRI), Inserm U1111, UCBL1, CNRS UMR 5308, ENS de Lyon, Université de Lyon, Lyon, France; 4 Laboratory of Cell Differentiation, Institute of Molecular Genetics of the Czech Academy of Sciences, Vídeňská 1083, Prague 4, 14220 Czech Republic

**Keywords:** Avian genome, Interferon signaling, Interferon regulatory factors, Gene loss

## Abstract

**Background:**

Interferon regulatory factors (IRFs) are a family of transcription factors with important functions in immunity. The genomes of most vertebrates encode ten IRF genes. IRF3 and IRF9 have key roles in interferon (IFN) induction and signaling. Most of our knowledge about the IFN pathways originates from the study of the mammalian IFN system, and the description of the corresponding avian components is not as complete. Both IRF3 and IRF9 were considered missing from the chicken genome and from the genomes of other avian species.

**Results:**

Here we describe multiple avian IRF3 and IRF9 genes, all with difficult GC-rich sequence context that prevented their earlier characterization. IRF3 orthologs are narrowly distributed and are present in the avian infraclass Palaeognathae. In contrast, IRF9 orthologs were found in most avian species, with the exception of the order Galliformes. In about half of the avian orders, IRF9 was located in noncanonical chromosomal positions, indicating past translocations. Phylogenetic analysis confirmed the correct orthology of all newly described IRFs. We further performed experiments using duck IRF9, confirming its role in the IFN pathway. IRF9 knockout in duck fibroblasts decreases the induction of IFN-stimulated genes (ISGs). Full induction of ISGs in duck cells requires both an intact IRF9 and a canonical IFN-stimulated response element. Lastly, intact IRF9 is needed for IFN-mediated protection of duck cells against the vesicular stomatitis virus-induced cytopathicity.

**Conclusions:**

The identification of avian IRFs fills an important gap in our understanding of avian immunology and brings new questions related to the evolution of the IRF family.

**Supplementary Information:**

The online version contains supplementary material available at 10.1186/s12915-025-02261-4.

## Background

The interferons (IFNs) are key cytokines orchestrating antiviral defense and immune regulation [1]. IFNs can be classified in three types based on sequence homology, receptor usage, and functional activity [2, 3]. Type I IFNs signal through the ubiquitously expressed type I IFN receptor (IFNAR) and activate a strong antiviral defense program. Type II IFN is mainly produced by immune cells and its main function is modulation of the adaptive immune response. Type III IFNs bind to distinct receptor (IFNLR) expressed mainly on epithelial cells but have similar activities as type I IFNs. In this work, we will mainly focus on the type I IFN activities.

Although interferon was originally discovered during work with embryonated chicken eggs [4], most of the subsequent molecular dissection of IFN-related pathways was performed in mammalian cells (reviewed in [5, 6]). Conceptually, the first stage of these pathways involves IFN induction, in which various sensor families detect viral products and via activation of several transcription factors induce production of these cytokines. The second stage is IFN signaling, during which secreted type I IFNs, including IFN-$\beta$ and multiple IFN-$\alpha$, interact with the type I IFN receptor and initiate a cascade of events, resulting eventually in the phosphorylation of signal transducers and activators of transcription (STAT) molecules. Various STAT complexes translocate to the nucleus and bind to genetic regulatory elements, like the IFN-stimulated response element (ISRE). This leads to the activation of hundreds of IFN-stimulated genes (ISGs) and the establishment of an antiviral state [7]. Importantly, both IFN pathway stages operate using transcription factors of the interferon regulatory factor (IRF) family. Two IRF family members, IRF3 and IRF7, are activated by upstream kinases and induce promoters of IFN genes. Subsequently, during the IFN signaling stage, IRF9 forms a trimeric complex with STAT1 and STAT2, also called IFN-stimulated gene factor 3 (ISGF3), which binds to ISRE. In contrast to the substantial knowledge about the mammalian IFN system, the understanding of the avian counterparts of transducers of IFN signaling is not as complete. Mainly in the last two decades, following the publication of the chicken genome sequence, many of the key components of the chicken innate immune defense have been described [8, 9]. A striking observation is the apparent lack of identified IRF3 and IRF9 in avian species [10, 11].

**Fig. 1:**
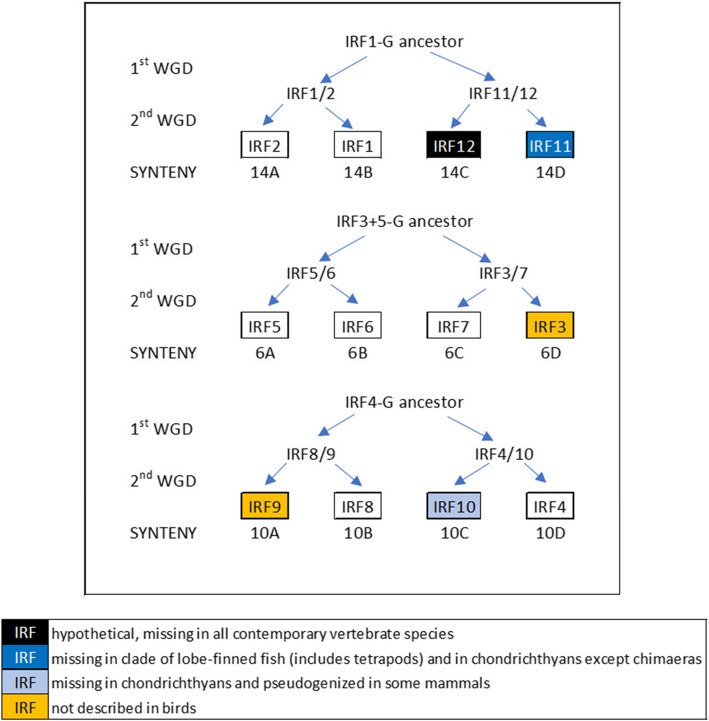
Synteny supports the hypothesis of the origin of the jawed vertebrate IRF gene family by two-fold genome duplication. The 2 WGD event led to the quadruplication of 17 gene groups (paralogons), each representing one chromosome of the pre-WGD chordate, into four quads designated A to D, resulting in 68 gene groups described and numbered from 1 A to 17D by [31]. Syntenic analysis indicates that IRF genes of each of the three IRF groups always belong to the same paralogon—paralogon 14 (IRF1-G: IRF1, 2, 11), paralogon 6 (IRF3 + 5-G: IRF3, 5, 6, 7), and paralogon 10 (IRF4-G: IRF4, 8, 9, 10)—and at the same time always to a different quad (**A**, **B**, **C**, **D**), indicating an origin from a single prevertebrate chordate gene for each group. Due to subsequent paralog loss, not all genes are present in all four copies in contemporary species. IRF4-G genes were classified as pertinent to paralogon 10 by [31]. For paralogon classification of IRF1-G and IRF3 + 5-G genes, see additional file (Additional file 1: Table S1)

In general, the IRFs of jawed vertebrates (Gnathostomata) are a family of eleven genes that encode transcription factors with important functions in immunity, hematopoietic differentiation, and other cellular processes [12]. Based on sequence similarity and synteny analysis, these eleven vertebrate paralogs are thought [13–15] to have evolved from three ancestral genes in an ancient chordate, which underwent two whole genome duplications (2 WGD) before the split into cartilaginous and bony fish vertebrate lineages in the early Paleozoic Era [16], giving three groups of genes: IRF1-G (which includes IRF1, 2, 11), IRF3 + 5-G (IRF3, 5,6, 7), and IRF4-G (IRF4, 8, 9, 10) (Fig. 1, Additional file 1: Table S1). Additional IRF paralogs might have evolved later, including those in vertebrate species that underwent a third or more successive genome duplications, like in teleost fish [17].

An unequal presence of IRF paralogs in different vertebrate species indicates that the 2 WGD event has been followed by the loss of some IRF genes. Nevertheless, a large number of amniote species have ten genes, IRF1 to IRF10, and most of the observed paralog absences, except IRF10 pseudogenization in some mammals, are likely the artifacts of incomplete genome sequences [18]. Therefore, the inability to discover IRF3 and IRF9 in avian species always seemed peculiar [10]. Some reports mentioned the presence of these two genes in the chicken, but that was always the result of confusion with other IRF paralogs. IRF3 was often confused with IRF7 (see [19]). The gene annotated as IRF9 in the chicken genome in GenBank and cited in some reports as such is actually the IRF10 gene described earlier on in the chicken [20].

It was previously shown that a subset of avian genes is encoded by sequences rich in G and C nucleotides (GC-rich) [21–24]. Such sequences are hard to identify with next-generation sequencing and also difficult to amplify using PCR. Therefore, such genes were often falsely reported as missing from avian genomes [25, 26]. We published and characterized several such “hidden” avian genes, including, for example, TNF-$\alpha$, FoxP3, LAT, and leptin [27–30]. In this work, we have focused on the discovery of avian IRF9 and IRF3. We report the identification of GC-rich IRF9 in multiple avian species and present an experimental characterization of duck (*Anas platyrhynchos*) IRF9. We also identified GC-rich IRF3 in paleognath birds.

## Results

### IRF3 genes were found only in genomes of basal avian infraclass of Palaeognathae

**Fig. 2:**
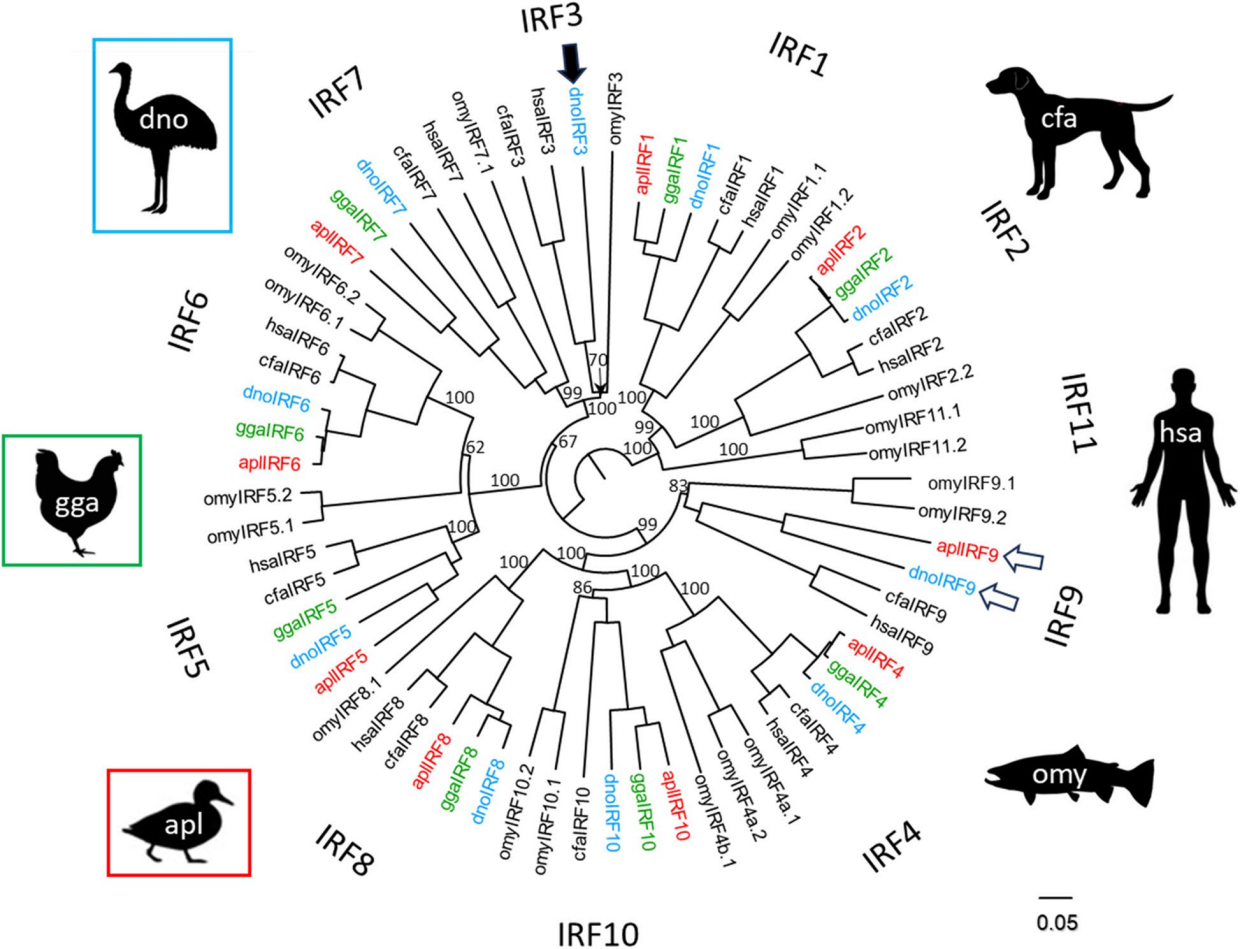
Position of avian IRF3 and IRF9 in the evolutionary tree of the IRF family. The black arrow and the white arrows indicate the respective positions of the avian IRF3 and avian IRF9 genes described in this report. Protein sequences of all IRF genes in six vertebrate species—rainbow trout (*Oncorhynchus mykiss*—omy), emu (*Dromaius novaehollandiae*—dno), chicken (*Gallus gallus*—gga), duck (*Anas platyrhynchos*—apl), human (*Homo sapiens*—hsa), and dog (*Canis familiaris*—cfa)—were compared. The fish family Salmonidae, which includes rainbow trout, has all 11 vertebrate IRF genes. Most of these IRF paralogs are present in multiple copies as a result of the multiplication of the original 11 genes in additional teleost and salmonid specific whole genome duplications. The IRF11 gene was not found in any tetrapod species. Humans lack a functional IRF10 gene. In the chicken, no IRF3 or IRF9 genes were identified and are likely missing. Protein sequences were extracted from NCBI protein databases or were assembled/predicted (emu IRF3 and IRF5, duck IRF5 and IRF9) from SRA database reads and genome contig sequences (see details in additional files; Additional file 1: Table S2 and S3). The sequences were aligned by ClustalX, a neighbor-joining (NJ) tree constructed and visualized by FigTree. Branch support values calculated by bootstrapping (10,000 replicates) are shown as percentages and only for central branches of the tree. The scale bar indicates the number of amino acid substitutions per site

A systematic search of avian sequences deposited in the National Center for Biotechnology Information (NCBI) public databases, including high-throughput sequencing repositories (Sequence Read Archive; SRA), resulted in the discovery of the first true avian IRF3 gene. The coding sequence of the gene found in the nucleic acid data of the emu (*Dromaius novaehollandiae*) was verified using a de novo assembly from the SRA RNASeq archives (Additional file 1: Table S2). The translated coding sequence of emu IRF3 was compared with amino acid sequences of other emu IRF paralogs and with sequences of complete sets of IRF genes from an additional two avian, two mammalian, and one teleost species (Fig. 2). The tree also included newly discovered duck and emu IRF9 genes, which will be discussed later. The phylogenetic tree showed the emu gene (black arrow) co-localized with the other previously characterized vertebrate IRF3 genes in one monophyletic group, confirming its IRF3 identity. The branching order of the tree was in general agreement with the syntenies shown in introductory Fig. 1 (except for the branching of IRF8 and IRF9, as well as the likely artificial clustering of trout IRF5 with IRF6 proteins), and also agreed with the IRF3 genes being the members of the higher order group, IRF3 + 5-G.

During the work on this manuscript, a new emu genome was released (GCF_036370855.1) that contained the IRF3 gene predicted by the NCBI annotation pipeline. This annotation mostly agrees with our coding sequence (CDS), except that the NCBI predicted protein sequence has the N-terminal extension and the CDS also contains what we consider to be an error—a break in the reading frame in exon 6 and a compensatory addition of one superfluous exon that is not supported by RNASeq data. The assembled emu IRF3 gene sequence was used to search for IRF3 genes in other avian genomes and transcriptomic data. The search resulted in the finding and the assembly of two complete coding sequences, one from the ostrich (*Struthio camelus*) and the other from the North Island brown kiwi (*Apteryx mantelli*), and one almost complete CDS from Darwin’s rhea (*Rhea pennata*) (Additional file 1: Table S2). Partial IRF3 coding sequences were also discovered in two additional bird species: chilean tinamou (*Nothoprocta perdicaria*) and great tinamou (*Tinamus major*) (Additional file 1: Table S2). These findings show that IRF3 genes are present in the genomes of all five extant orders of paleognath birds (Struthioniformes, Rheiformes, Tinamiformes, Apterygiformes, and Casuariiformes).

**Fig. 3:**
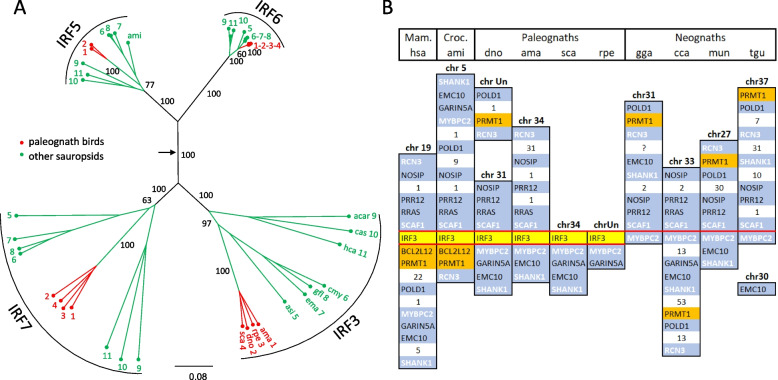
Phylogenetic and syntenic analyses of avian IRF3 genes.** A** Avian IRF3 sequences co-localize with IRF3 sequences from non-avian sauropsid species in a single clade in the phylogenetic tree. IRF3 protein sequences of four avian species (ama—*Apteryx mantelli*, kiwi; dno—*Dromaius novaehollandiae*, emu; rpe—*Rhea pennata*, rhea; sca—*Struthio camelus*, ostrich) and seven non-avian sauropsids (asi—Alligator sinensis, Chinese alligator; cmy—*Chelonia mydas*, sea turtle; gfl—*Gopherus flavomarginatus*, tortoise; ema—*Emydura macquarii*, side-neck turtle; acar—*Anolis carolinensis*, anole; cas—*Candoia aspera*, boa; hca—*Hemicordylus capensis*, crag lizard) were aligned with sequences of closely related paralogs (IRF5, 6, and 7) from the same or closely related species (ami—*Alligator mississippiensis*, American alligator). The sequences were either assembled/predicted from NCBI SRA or WGS archives or were identified among translated sequences in databases of NCBI assembled genes, and are all listed in an additional file (Additional file 1: Table S3). The NJ tree constructed from the alignments revealed separation of the sequences into four clusters corresponding to four IRF genes. Species in the IRF5, IRF6, and IRF7 cluster are designated using the numbers shown side by side with species abbreviations in the IRF3 cluster. The black arrow indicates the position of the root, based on the hypothesis of the origin of the IRF3 + 5-G genes by 2 WGD. The phylogenetic tree was constructed by ClustalX. Branch support values calculated by bootstrapping (1000 replicates) are shown as percentages, and only for central branches of the tree. The scale bar indicates the number of amino acid substitutions per site. **B** Conserved genomic position of IRF3 in paleognaths and the absence of the IRF3 gene in the syntenic location in neognathes. Protein-coding genes and their positions on both sides of the IRF3 gene (where available) in the four species of paleognathes mentioned in the legend for panel A were compared with the positions of the same genes in the genomes of the human (hsa), American alligator (ami), and four species of neognathes (gga—*Gallus gallus*, chicken; cca—*Cuculus canorus*, cuckoo; mun—*Melopsittacus undulatus*, budgerigar; tgu—*Taeniopygia guttata*, zebra finch). Other abbreviations: Mam.—mammals, Croc.—crocodiles. The genomes used are listed in an additional file (Additional file 1: Table S6). Contigs not assigned to any chromosome are designated as chrUn. IRF3 genes are emphasized using a yellow background and red cell boundaries. Genes found immediately on the 3’ side of IRF3 in the human and alligator and missing or relocated in birds are shown using an orange background. IRF3 genes or the locations where they are missing in neognathes are in all species surrounded by ohnologs from the group 6D (group defined by [31]; ohnologs shown in white ink). The presence of these ohnologs indicates the preservation of the macrosynteny established by the second WGD. When some protein-coding genes were omitted from the figure, the gaps where they would be placed were marked by the number of genes omitted

Emu, ostrich, kiwi, and rhea genes were studied further in more detail. Protein sequences of these four genes were aligned with the sequences of the matching avian paralogs from the IRF3 + 5-G group (IRF5, 6, 7), as well as with IRF proteins from several other, non-avian reptiles, and a phylogenetic tree was constructed (Additional file 1: Table S3, Fig. 3A). Monophyletic clustering of avian IRF3 sequences with IRF3 proteins of other reptiles confirmed their IRF3 orthology. Syntenic analysis suggested that IRF3 genes in all four avian species are located at a conserved position on a microchromosome that represents an equivalent of chicken chromosome 31 (Fig. 3B). In avian species, as well as in the human and the Chinese alligator, the IRF3 gene is surrounded by the genes that include MYBPC2, RCN3, SCAF1, and SHANK1, which belong to the group of ohnologs 6D. Ohnologs are gene duplicates that originated by whole genome duplication and were named in honor of Susumo Ohno, who was the first to suggest a role of genome duplications in the evolution of the vertebrate genome. The group 6D is one of 68 syntenic groups defined by Lamb [31]. These syntenic groups emerged as quadruplicates of the gene content of 17 chromosomes of the pre-2 WGD chordate. Genes of the group 6D also surround IRF3 in the genomes of the great white shark (*Carcharodon carcharias*), coelacanth (*Latimeria chalumnae*), and caecilian (*Microcaecilia unicolor*). The avian IRF3 gene, therefore, keeps the same macrosynteny that was established after the second WGD. Microsynteny in the sense of exact gene order changed, however, during evolution. While SCAF1 neighbors the 3’ side of IRF3 in most jawed vertebrate species we examined, BCL2L12 resides on the 5’ side, followed by PRMT1 in the human and alligator, while in birds MYBPC2 is located there instead.

Sequence comparison of emu, ostrich, kiwi, and rhea IRF3 genes with genes of other amniote species showed both conserved and distinct features of these avian genes. The IRF3 coding sequences in all four birds were surprisingly similar (91%, 90%, 89%, 90%, 90%, and 89% of identity between emu-ostrich, emu-kiwi, emu-rhea, ostrich-kiwi, ostrich-rhea, and kiwi-rhea), despite an estimated divergence of these species occurring approximately between 60 and 80 million years ago [32]. All these coding sequences were GC-rich with GC content 76–77%, and the proteins they encode had an increased percentage of the four amino acids (A, G, P, R) with GC-rich codons (Additional file 2: Fig. S1, Additional file 1: Table S3). High GC and AGPR-codon contents of avian genes contrasted with lower values in alligators and turtles and even lower percentages in lepidosaurs, mammals, and acanthodian fish.

Alignment of protein sequences of four paleognath species with proteins from an alligator, turtle, and three mammalian species (Additional file 2: Fig. S2) showed that five conserved tryptophans, a signature feature of the IRF DNA binding domain, were present in all aligned sequences. Similarly, two serines in the IRF3 C-terminus instrumental for the activation of IRF3 by phosphorylation were conserved as well [33]. Compared to the seven exon structure of mammalian IRF3 genes, avian genes have nine exons, similar to the genes of non-avian archelosaurs (crocodiles + turtles). Interestingly, both our and the genomic version of emu IRF3 CDS contain an in-frame extension of the second exon via a four-fold repetition of amino acid codons at the 3’ boundary of the exon. Sequences of the other three paleognath birds did not have these in-frame extensions, indicating that this structural variation is likely limited to the emu and possibly species closely related to it.

In contrast to paleognath birds, no IRF3 was found in 8 high-quality genomes representing a wide range of neognath species (Additional file 1: Table S4). In all cases, these genomes contained the SCAF1 gene, which is located on the chromosome directly beside IRF3 in almost all reasonably complete genomes of non-avian jawed vertebrates that we examined, as well as in the genomes of the emu and kiwi. The presence of SCAF1 suggests that these neognath genomes are not missing sequences of chromosomal regions in which IRF3 would be located if conserved syntenies were preserved. The synteny analysis also indicated that the space between SCAF1 and MYBPC2 where IRF3 resides in paleognathes is not occupied in the four neognath species examined by any protein-coding gene (Fig. 3B). Further searching in the default nucleotide (nr/nt) GenBank Blast databases retrieved 15 neognath protein sequences annotated as IRF3 (Additional file 1: Table S5). However, all these genes clustered in the evolutionary tree with IRF7 genes and not with IRF3 (Additional file 2: Fig. S3). Therefore, IRF3 genes seem to be missing in all but paleognath birds.

### Identification and sequence characterization of duck IRF9

Similar searches of genetic databases focused on avian IRF9 genes recovered multiple hits with high GC content. Such sequences were usually highly incomplete and were present on unplaced genomic contigs. In some cases, e.g., zebra finch (*Taeniopygia guttata,* XM_041711703) and some other song birds, NCBI automated computational analysis predicted the presence of an IRF9-like gene. Using the *T. guttata* sequence as a probe in blast searches, we detected a nearly complete IRF9 in the wild duck (*A. platyrhynchos*) genomic contig NOIJ01000842. Publicly available RNASeq datasets (e.g., NCBI SRA study PRJNA509092) were then used to fill the gaps and assemble a full coding sequence of duck IRF9 (Additional file 1: Table S2).

The protein sequence of the newly identified duck IRF gene was compared with sequences of other duck IRF paralogs and with complete sets of IRF genes from an additional two avian, two mammalian, and one teleost species. A phylogenetic tree showed the duck gene co-localized with the other previously characterized IRF9 genes, as well as with an emu sequence annotated as IRF9-like in one monophyletic group, confirming its IRF9 identity (Fig. 2). The branching order of the tree validates the IRF9 genes as members of the higher order group, IRF4-G.

**Fig. 4:**
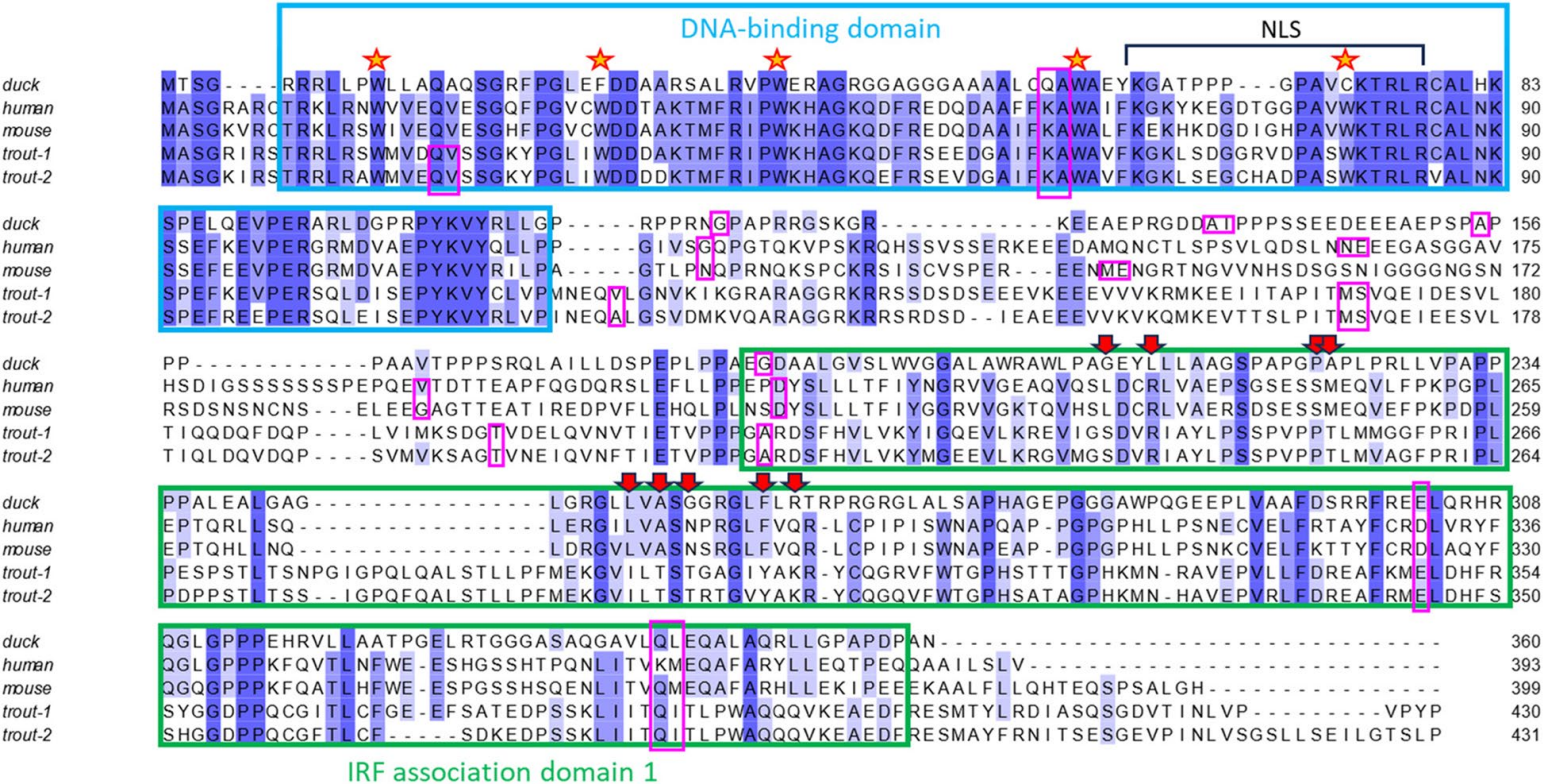
Multiple alignment of IRF9 proteins. Principal functional domains, DBD and IAD1, are designated by rectangles with blue and green outlines, respectively. The positions of five conserved tryptophans in the DBD, the prominent feature of IRF proteins, are marked by yellow stars with red boundaries. The DBD also contains a nuclear localization signal (NLS). The red arrows in the IAD1 domain designate the amino acids important for the interaction of human IRF9 with STAT2. The amino acids with codons split by introns are indicated by rectangles with pink outlines. If any intron splits CDS exactly between two codons, both of the encoded amino acids are included in the rectangle. The other sequence features are discussed in the main text. The sequences were aligned by ClustalX and the alignment visualized using Jalview 2.11.3.3. Used protein sequences are listed in an additional file (Additional file 1: Table S3)

The multiple protein sequence alignment allowed us to compare functional domains and conserved amino acid positions between duck IRF9 gene and the best studied vertebrate IRF9 genes of human, mouse, and fish (Fig. 4). The sequence of duck IRF9 was generally similar to protein sequences from the other species, but surprisingly exhibited a high level of diversity in several regions. In a large portion of the DNA binding domain (DBD) and in several regions of the IRF association domain (IAD1), the sequences of the fish and mammalian proteins were more similar to each other than either of them was to the avian protein. Also, two of the conserved tryptophans in DBD were replaced in the duck IRF9 by different amino acids—phenylalanine and cysteine. Similar replacements were described before in other IRF proteins, but are relatively rare [13]. Only three of the nine amino acids important for the interaction of human IRF9 with STAT2 [34] are conserved in duck protein.

Duck IRF9 CDS is split into eight exons, as is typical for other mammalian and avian genes of the IRF4-G group. Its GC content is 80%, which is in contrast with mammalian and other vertebrate IRF9 orthologs whose GC content is usually around 60%. Exon boundaries (pink outlined rectangles in Fig. 4) in the CDS are highly conserved inside DBD and IAD1, and more variable in the central linker region. The first two exons contain the sequence of the conserved DBD and short N-terminus, while the last three encode IAD1 and the C-terminus of variable length. The three exons in the middle (exons 3–5) encode the DBD-IAD1 linker with a low level of sequence conservation. Trout IRF9 genes have the region equivalent to the first duck/mammalian coding exon split into two exons. The introns of the duck IRF9 gene are relatively short, in total only 2.5 times longer than the CDS. Such compact gene structure is typical for most vertebrate IRF genes.

### IRF9 genes are part of the gene sets of most avian species

Further iterative rounds of blast queries using the complete duck IRF9 gene sequence and multiple newly identified avian IRFs were done against NCBI databases. These searches for IRF9 genes in other avian species resulted in a substantial collection of 38 genes, including 34 full length and 4 partial CDS from 37 species (Table 1). These species represent 16 avian orders, including the species-rich song birds (Passeriformes), suggesting a pan-avian distribution of the IRF9 gene. Additional partial IRF9 sequences not indicated in the table were found in other song birds, but were not further analyzed. Interestingly, no IRF9 sequences were found in Galliformes (landfowl) despite the availability of a number of highest quality genomes from this bird order.

**Table 1:**
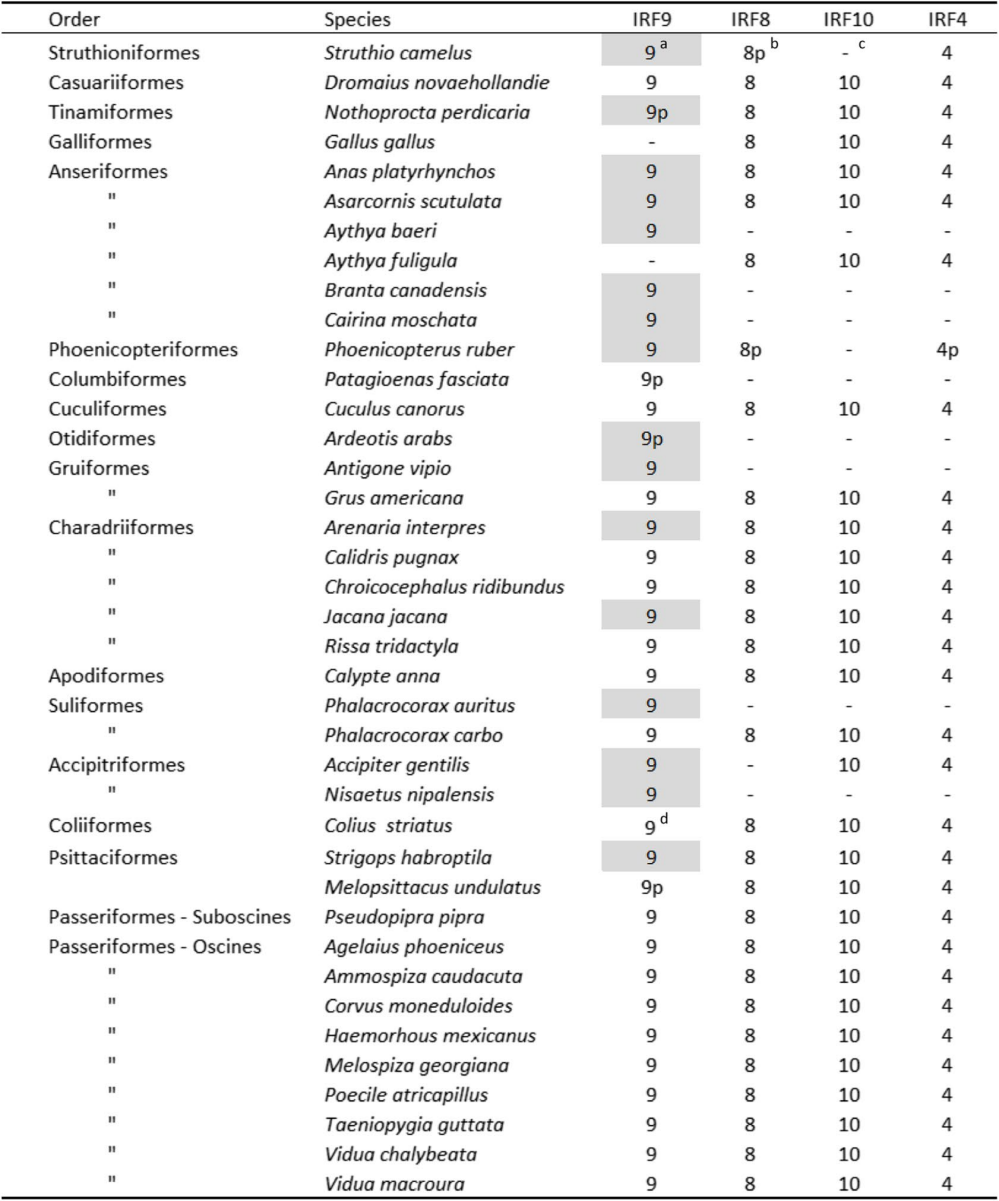
Avian IRF9 genes identified. Sequences of matching paralogs of the IRF4-G group were also retrieved in species with available annotated genomes. All sequences are included in an additional file (Additional file 1: Table S3)

^a^A number without a p suffix indicates a full-length coding sequence identified for the given IRF gene. A gray background specifies the genes whose sequences were assembled/predicted from NCBI SRA RNASeq and whole genome sequencing contigs databases. Other genes were identified among translated sequences in databases of NCBI predicted genes

^b^ The suffix p indicates a partial sequence

^c^ A dash indicates that the gene was not found in NCBI databases for that species. For most of these genes (except for IRF9 in *Gallus gallus*), assembly from SRA archives was not attempted

^d^ There are two IRF9 genes on the same chromosome in that species

**Fig. 5:**
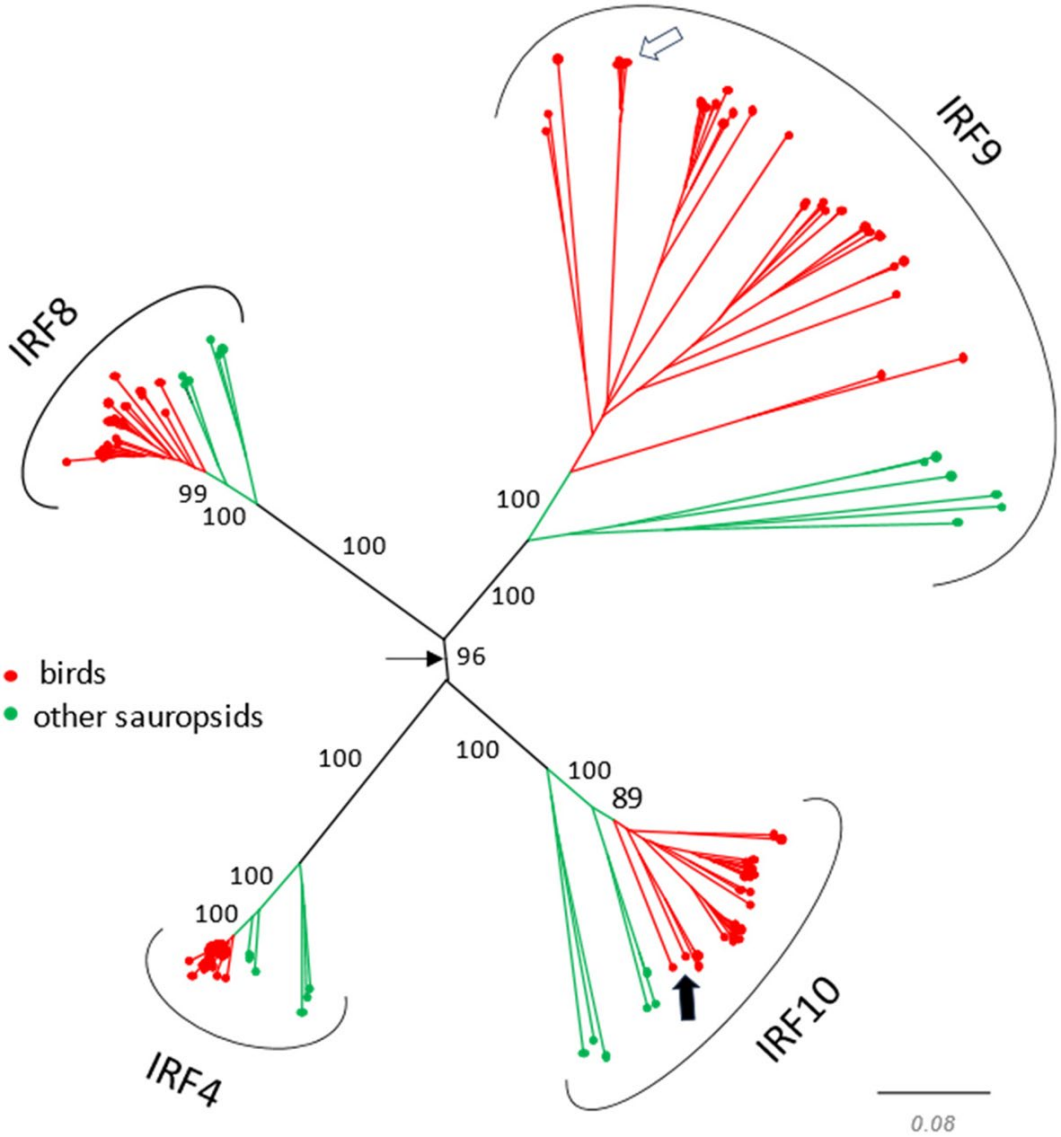
Avian IRF9 protein sequences form a single clade in the phylogenetic tree. Thirty-four avian IRF9 genes with complete and two with partial CDS from those genes listed in Table 1 are shown in the tree. IRF4, 8, and 10 sequences from the same or closely related species (if available) and sequences from selected non-avian sauropsids are also present in the tree. The tree also contains IRF4, 8, and 10 of the chicken, in which the IRF9 gene was not found. The sequences were either assembled/predicted from NCBI SRA or WGS archives, or were identified among translated sequences in databases of NCBI assembled genes, and are all listed in Table 1 and/or in an additional file (Additional file 1: Table S3). The thin black arrow indicates the position of the root based on the hypothesis of the origin of IRF4-G genes by 2 WGD. The white arrow indicates the position of the anseriform IRF9 proteins, including duck IRF9. The thick black arrow shows the location of the chicken IRF10 protein, annotated in NCBI genomic databases as IRF9. The tree shown in this figure is also shown in the form of a circular cladogram with species labels included in an additional file (Additional file 2: Fig. S4). The NJ tree was constructed from protein sequence alignment by ClustalX. Branch support values calculated by bootstrapping (1000 replicates) are shown as percentages, and only for central branches of the tree. The scale bar indicates the number of amino acid substitutions per site

The phylogenetic tree of the newly described avian IRF9 genes was constructed to establish their correct orthology (Fig. 5, Additional file 2: Fig. S4). Avian IRF9 protein sequences were compared with the sequences of their matching paralogs from the IRF4-G group (IRF4, 8, 10) (Table 1). Also, complete sets of four IRF4-G genes from several non-avian reptiles were added to the group of analyzed genes (Additional file 2: Table S3). Monophyletic clustering of the avian IRF9 sequences confirmed their correct identification. Again, the chicken IRF10 gene (thick black arrow) annotated in the NCBI database as IRF9 clustered with IRF10 genes and not with IRF9 genes.

### Conserved syntenic location of duck IRF9 contrasts with noncanonical chromosomal positions of IRF9 in a majority of avian species

**Fig. 6:**
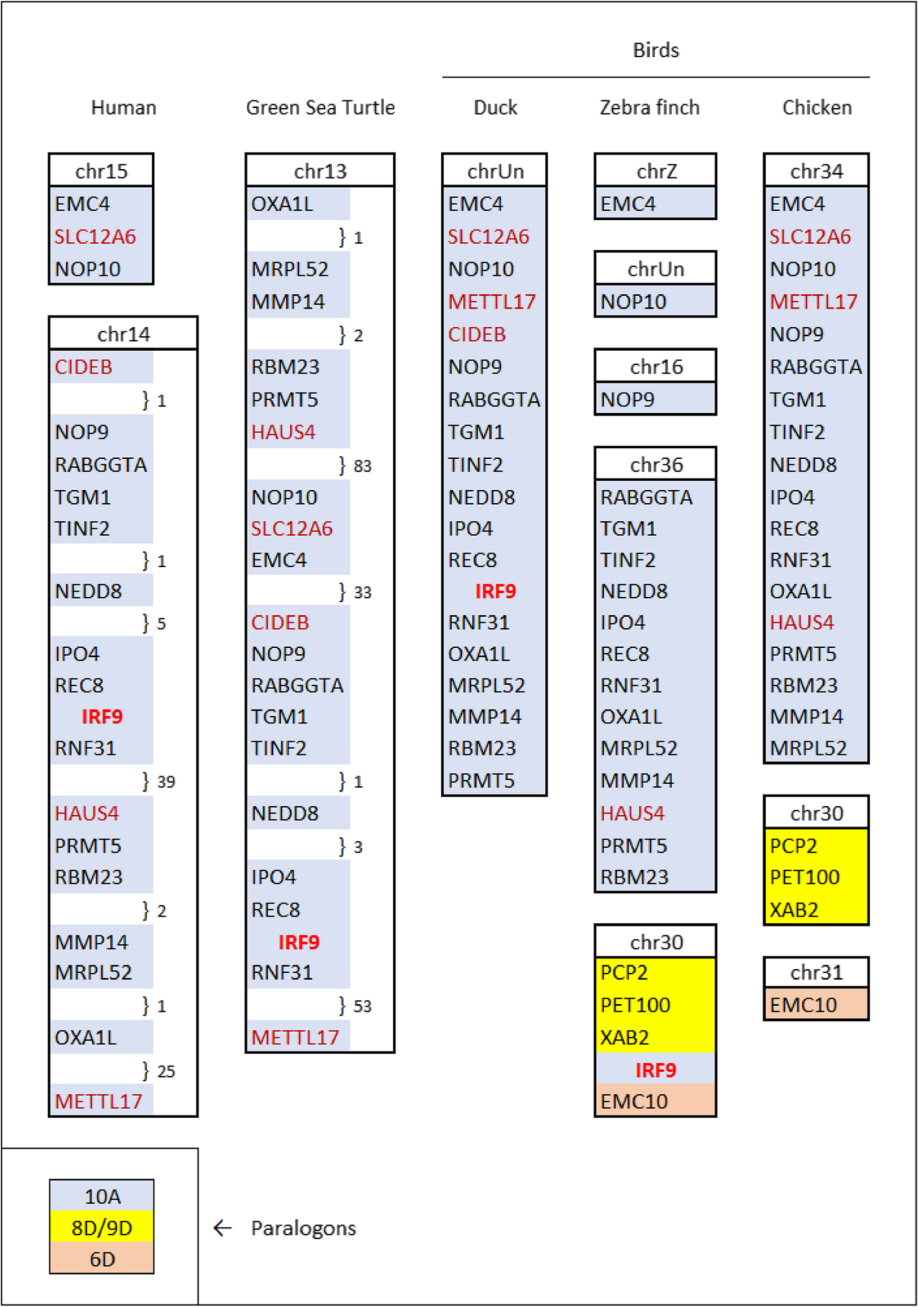
Conserved genomic position of duck IRF9, the gene relocation in song birds, and the absence of the IRF9 gene in galliform birds. Protein-coding genes and their positions identified in duck contig NOIJ01000842.1 on both sides of the IRF9 gene were compared with turtle and human genes (left side of the figure), as well as zebra finch and chicken genes (on the right). Chromosomes are numbered based on the current default genomes in the NCBI database, except in the chicken, where numbering follows the conventions of Ggswu assembly (huxu breed). The duck contig was not localized to any chromosome (chrUn). The genomes used are listed in an additional file (Additional file 1: Table S6). The positions of duck and chicken genes were determined by tblastn and the integrity/completeness of their coding sequence was not verified. The same 20 genes are shown in the turtle and human. Genes not found in the duck (HAUS4), in the finch (CIDEB, METTL17, SLC12 A6) or chicken (CIDEB, IRF9) are emphasized by red or purplish ink. When some protein-coding genes were omitted from the figure, the gaps where they would be placed were marked by the number of genes omitted. Additional genes surrounding the relocated IRF9 are shown in the finch, and corresponding genes are also shown in the chicken. The colored background indicates paralogon affiliation (see legend in lower left corner). The paralogon affiliation was estimated based on the association by synteny in multiple vertebrate genomes, with genes ascribed to specific paralogons by Lamb [31]

Gene synteny in vertebrates is conservative and provides an important tool for the assessment of gene orthology [16, 35]. To establish if duck IRF9 is indeed co-localized on a chromosome with the same genes as in other vertebrate species, we analyzed the gene content of the longest available duck genome contig containing IRF9 (Fig. 6). All of the genes, including IRF9, found on that contig were also found on a single chromosome (chr13) in the green sea turtle (*Chelonia mydas*). In the human genome, 85% of these genes are positioned on the IRF9-containing chromosome, chr14. Duck IRF9, therefore, resides in an evolutionarily conserved chromosomal position. Similarly, at least some of those genes surrounding duck IRF9 were detected in the immediate vicinity of an IRF9 ortholog in the chromosomal sequences of emu (*Dromaius novaehollandiae*), common cuckoo (*Cuculus canorus*), whooping crane (*Grus americana*), great cormorant (*Phalacrocorax carbo*), eurasian goshawk (*Accipiter gentilis*, recently renamed as *Astur gentilis*), and budgerigar (*Melopsittacus undulatus*). These findings suggest that IRF9 is also located in a conserved syntenic position in six other avian orders: Casuariiformes (emus and cassowaries), Cuculiformes (cuckoos and others), Gruiformes (cranes, rails and allies), Suliformes (gannets, cormorants and allies), Accipitriformes (hawks and eagles), and Psittaciformes (parrots and others), in addition to the order Anseriformes (ducks, geese and swans). Song birds (Passeriformes), however, which represent some 60% of avian species, have the IRF9 gene located in a noncanonical position. The zebra finch (*Taeniopygia guttata*) IRF9 is on chromosome 30, while orthologs of the 13 genes that are close neighbors of duck IRF9 are instead on the zebra finch chromosome 36 (Fig. 6). The same noncanonical position is also occupied by IRF9 in other song bird species, including all of the species shown in Table 1.

Gene syntenies in vertebrates are to a large extent predetermined by the two-fold whole genome duplication (WGD) that affected their ancestors in the older paleozoic [16]. Genes of jawed vertebrates can be divided into 17 groups—paralogons, each representing one chromosome of the pre-WGD chordate. Each paralogon is present in the genome in four copies designated A to D, resulting in 68 gene groups identified by Lamb [31] and numbered from 1 A to 17D. These gene groupings are surprisingly stable and still recognizable in genomes of contemporary vertebrates. The IRF9 gene is part of paralogon 10 A and, by association, the other genes on the duck IRF9 contig are as well. In contrast, a majority of the genes on the zebra finch IRF9 chromosome belong to paralogons 8D/9D. Genes surrounding the zebra finch IRF9 on chromosome 30, therefore, have a completely different evolutionary syntenic origin than those of the duck. Other noncanonical positions of IRF9 were also found in five other avian orders. In each of these five cases, IRF9 is positioned in a distinct genomic context and is surrounded by genes associated with different paralogons. In Tinamiformes (tinamous), these are paralogons 1D/10D, in Columbiformes (pigeons and others) 5 C, in Charadriiformes (shorebirds and allies) 3 C, in Apodiformes (swifts and hummingbirds) 6 C (a phenomenon likely limited to hummingbirds only), and in Coliiformes (mousebirds) 10 C.

Galliform birds are the only avian order in which genes of the paralogon group 10 A, including the closest IRF9-associated genes in the duck, were found but repeated attempts to find IRF9 failed. The chicken chromosome 34, where genes of group 10 A reside, as well as other chicken chromosomes except W, were sequenced in their entirety from telomere to telomere [36]. Nevertheless, no traces of the IRF9 sequence could be located in this or in any other chicken genome assemblies. The chromosomal locations in the chicken genome to which IRF9 was translocated in song birds (Fig. 6) and the five other avian orders mentioned above are empty as well. Searches in genomes of other galliform birds also failed.

### Duck IRF9 is essential factor for IFN signaling

**Fig. 7:**
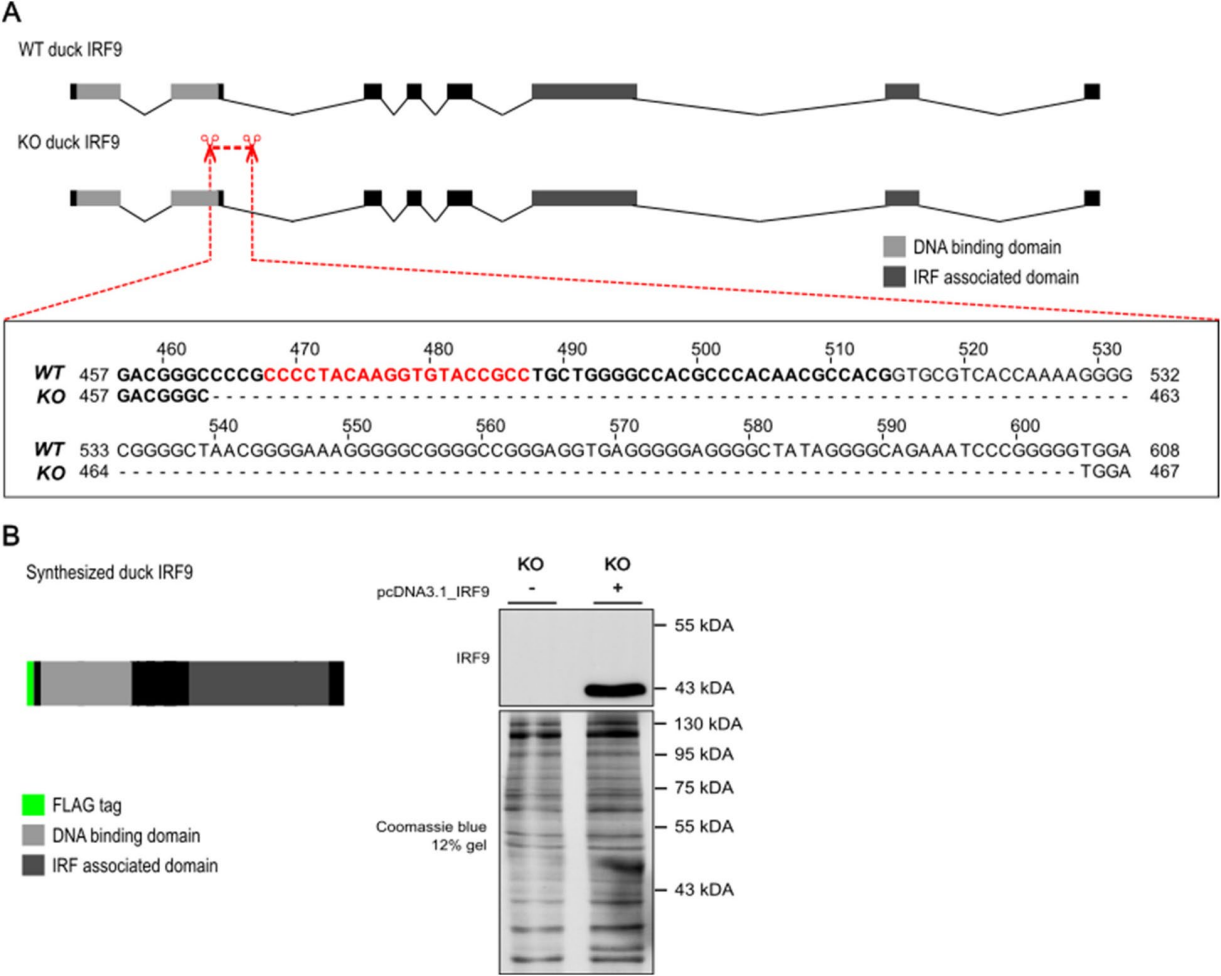
Duck IRF9 knockout and in vitro synthesized construct. **A** A schematic depiction of duck IRF9 exon structure in wild type (WT) and knockout (KO) cells. Only protein-coding parts of the first and last exons are shown. The position of the deletion in the KO clone is highlighted and expanded in the sequence below. Bold text represents the exon sequence and regular font the intronic part of the gene. Red font represents the position of gRNA used to target the CRISPR/Cas9 complex. Numbering of the alignment starts at the start codon of the CDS and includes introns. **B** The scheme of the protein encoded by the in vitro synthesized duck IRF9, tagged with a FLAG tag at the N-terminus; a western blot of lysate from KO cells transiently transfected by the dIRF9 expressing plasmid, detected by an anti-FLAG antibody

We chose duck IRF9 for further experimental work, aiming to assess its role in the IFN signaling pathway. Previously, we generated an immortalized cell line based on duck embryonal fibroblasts (see Methods). By using CRISPR/Cas9 targeted modification in this cell line, we prepared a duck IRF9 knockout cellular clone (KO) (Fig. 7) and compared its responsiveness to duck IFN-$\alpha$ with a wild type (WT) clone. As a readout of IFN signaling, we measured the mRNA induction of two duck ISGs—interferon induced protein with tetratricopeptide repeats 5 (IFIT5) and myxovirus resistance protein (Mx). For these experiments, we also prepared a duck IRF9 (dIRF9) expression plasmid with a N-terminal flag tag. As this construct was prepared by commercial gene synthesis, the codons were optimized to avoid GC-rich stretches in the IRF9 sequence, but to fully conserve the protein sequence (Additional file 2: Fig. S5).

**Fig. 8:**
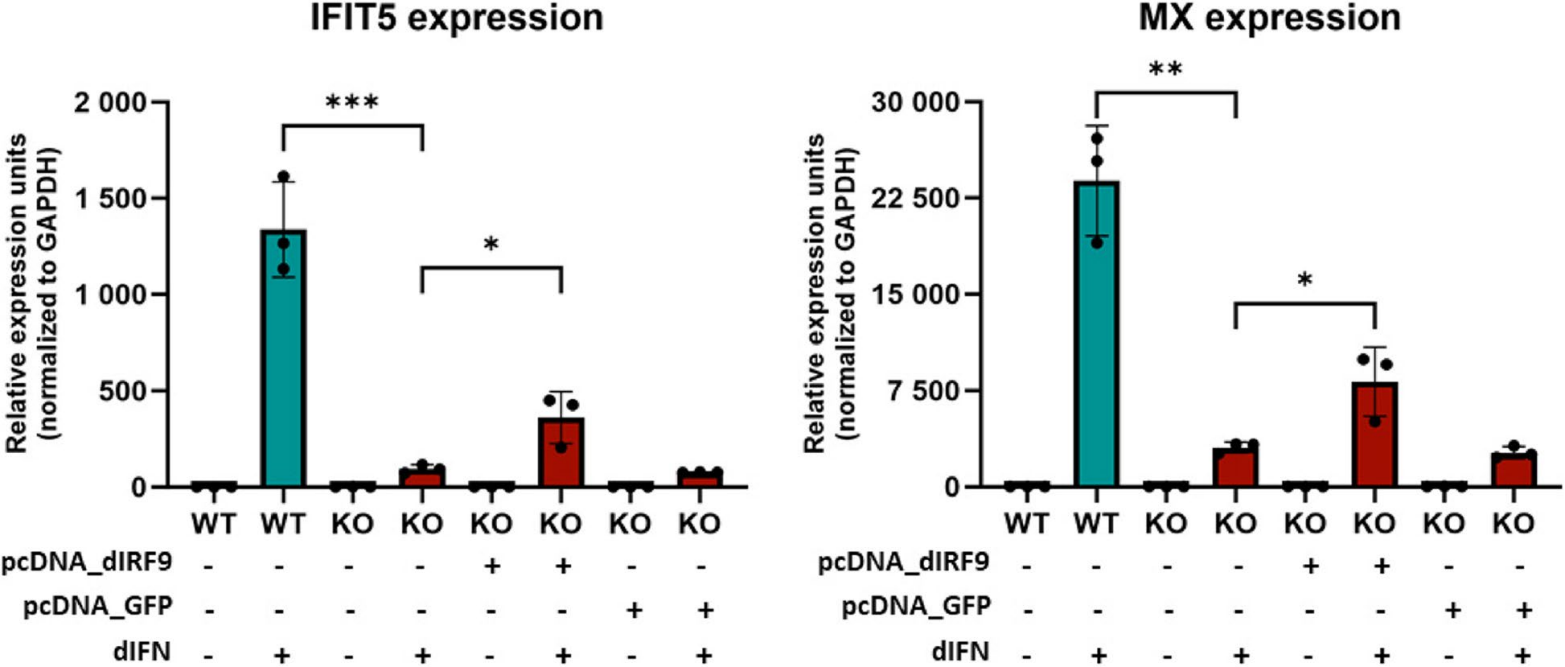
Expression of ISGs after IFN stimulation in IRF9 knockout cells. WT and KO cells were either transfected with plasmid containing in vitro synthesized duck IRF9 (dIRF9) or with green fluorescent protein (GFP) expressing plasmid used as a negative transfection control, or they remained untransfected. Relative mRNA expression (normalized to GAPDH expression) of interferon stimulated genes IFIT5 and Mx after duck IFN (dIFN) treatment was measured by quantitative PCR. Graphs and nested t-tests were done in GraphPad Prism 10. *p ≤ 0.05; **p ≤ 0.01; ***p ≤ 0.001

Duck IFN caused high induction of both IFIT5 and Mx mRNA expression in WT cells (Fig. 8). This induction was strongly decreased in duck IRF9 KO cells. Further, transfection of the dIRF9 expression plasmid into KO cells partially restored the ISG expression. The partial restoration of IFN sensitivity is consistent with the fact that IRF9 was delivered by transient transfection, and therefore not all cells were targeted. Alternatively, the protein level of ectopically expressed IRF9 might not be optimal. Overall, these results confirm that duck IRF9 is essential for the IFN signaling pathway.

### Both IRF9 and intact ISRE are required for high ISG induction

**Fig. 9:**
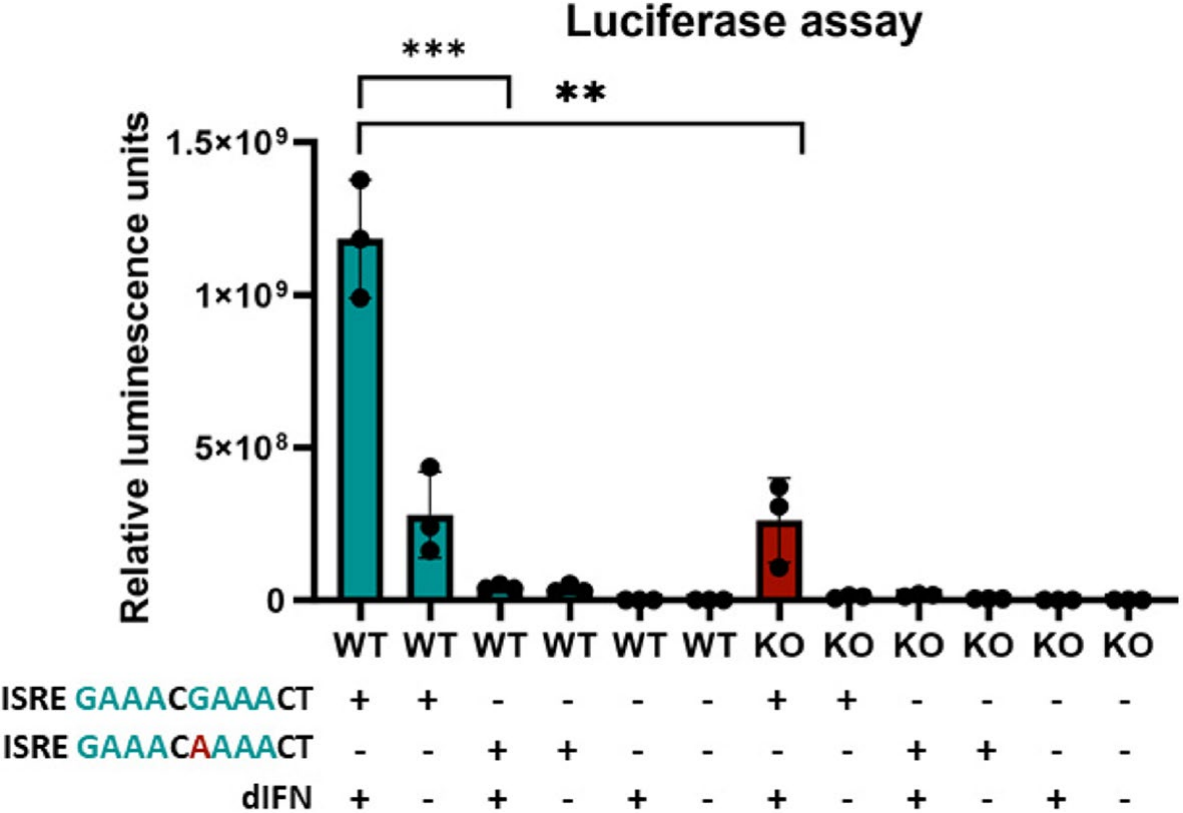
Duck IRF9 and consensus ISRE sequence, needed for expression of ISGs. Duck IRF9 WT and KO cells were transfected with pNL1.2 NLucP plasmids containing an ISRE-dependent tetherin promoter region and the NanoLuc luciferase reporter gene. One plasmid had a promoter region containing consensus ISRE sequence GAAACGAAACT. A second plasmid had a promoter region containing the ISRE sequence with a point mutation G > A—GAAACAAAACT (nucleotide substitution highlighted in red font). Upon transfection with ISRE constructs and dIFN stimulation for 24 h, the luciferase activity was determined. Graphs with a nested t-test were done in GraphPad Prism 10. **p ≤ 0.01; ***p ≤ 0.001

Next, we asked whether the ISG induction pathway in duck cells requires intact ISRE. For this purpose, we used a luciferase reporter construct preceded by the promoter region of the duck tetherin gene. Tetherin (also called bone marrow stromal antigen 2; Bst2) is a highly IFN-induced avian gene, which we previously characterized in chicken and other avian species [37, 38]. We generated two variants of the tetherin-luciferase construct: (i) with a native ISRE sequence (complying with the ISRE consensus; [39]) and (ii) a mutant with a key guanine changed to adenine (Fig. 9). Upon transfection into WT duck cells followed by dIFN addition, the construct with intact ISRE drove high luciferase reporter expression. Only low luciferase activity was detected in the construct with mutant ISRE. Consistent with previous experiments, expression of the ISRE-driven reporter was lower in IRF9 KO cells. These experiments show that ISG expression in duck is dependent on intact ISRE and that both intact ISRE and functional IRF9 are required for full IFN-induced ISG induction.

### IRF9 is necessary for IFN-dependent protection against VSV cytopathic effect

**Fig. 10:**
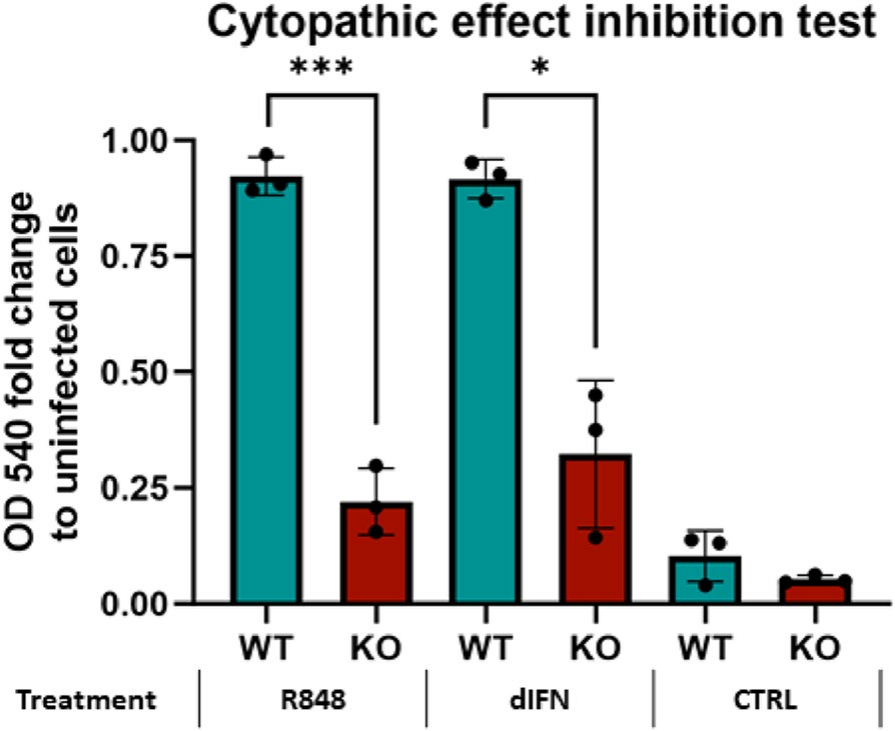
Role of IRF9 in protection of duck cells against the cytopathic effect of VSV after IFN stimulation. Duck IRF9 WT and KO cells were incubated with the following: a medium with a supernatant from R848-stimulated duck spleen cells (positive control), a duck interferon supernatant (dIFN) produced in HEK293 T cells, or a control supernatant (CTRL). After 24 h, cells were infected with VSV and 24 hpi, and after the addition of neutral red for 2 h, OD was measured using an ELISA reader at a wavelength of 540 nm, indicating cell survival. WT cells showed a significantly higher survival after incubation with R848 and dIFN compared to KO cells. A Graph and Welch’s t-test were done in GraphPad Prism 10. *p ≤ 0.015; **p ≤ 0.01; ***p ≤ 0.001

The importance of duck IRF9 in the IFN signaling pathway culminating with ISG induction was established above. We also assessed the role of IRF9 in protection against virus infection. To achieve this, we used a well-established model of vesicular stomatitis virus (VSV) induced cytopathic effect. The cytopathic effect can be prevented by IFN treatment in the context of a functional IFN signaling pathway [40]. This has also been used as a basis for determining IFN activity [41]. We compared the ability of IRF9 KO and WT cells to be protected against VSV-mediated cell killing by duck IFN. As a positive control, we used a supernatant from duck splenocytes stimulated with R848 (a toll-like receptor ligand), which induces multiple cytokines, including interferons. Indeed, while the WT cells could be protected by IFN addition against the VSV cytopathicity, the IRF9 KO cells showed significantly lower survival (Fig. 10).

## Discussion

In this work, we identified two avian interferon regulatory factors, IRF3 and IRF9. Both were not characterized before and were considered missing in the chicken and by extension in all birds. All the newly identified IRF3 and IRF9 orthologs are GC-rich. Therefore, their delayed identification is in line with the previous cases of “hidden genes” described by us and by others [25, 26]. Despite the new identifications, our searches showed that the orthologs of the two IRFs are not present in all avian species. IRF3 orthologs are more narrowly distributed and were found only in the basal avian infraclass Palaeognathae, which includes ostriches, kiwis, etc. In contrast, IRF9 orthologs are present in almost all avian species, with the notable exception of the order Galliformes, which includes the domestic chicken.

It remains formally possible that even in the instances in which we currently predict an IRF gene loss, the respective IRF ortholog could, in fact, still be present (e.g., IRF9 in galliform birds or IRF3 in Neognathae). This could happen because the extremely difficult DNA sequence of these genes (high GC-content, complex secondary structure) cannot be processed by any current sequencing technology. Alternatively, the sequence divergence of the respective IRF ortholog could be so large that it would be missed by our homology-based searches. Indeed, avian IRF9 gene sequences diverged more extensively than the sequences of any other IRF. Nevertheless, given the largely unbiased nature of new long-read sequencing platforms, especially the Oxford Nanopore [42], and the sensitivity of blast-based searches even for distant relatives (Additional file 1: Table S4), we consider the existence of such hidden IRFs rather improbable.

Both IRF3 and IRF9 genes from avian species have high GC content and corresponding change in amino acid composition. There is an increase in amino acids encoded by GC-rich codons, as shown before for this type of GC-rich avian genes [26]. Avian IRF9 has even higher GC content than IRF3, and this content decreases when IRF9 becomes translocated to non-dot chromosomes (Additional file 2: Fig. S1).

The IRF3 identified only in the infraclass of Palaeognathae was always located on the chromosome next to SCAF1, in the syntenic position conserved at least from the last common ancestor of all contemporary gnathostomian vertebrates. In neognath species, that gene position beside SCAF1 was found empty. Similarly, IRF9 genes in anseriform duck and in representative species of many other avian orders were located in an evolutionarily conserved position, i.e., between RNF31 and REC8. In galliform birds, however, no traces of IRF9 were found between these two genes, nor in any other place in the genome. Surprisingly, in various species of about half of the avian orders studied, the IRF9 was found located in several noncanonical chromosomal positions, indicating past translocations in the course of avian evolution. These translocations were not the relatively common intrachromosomal gene reshufflings, but transfers to a different chromosomal context, indicated by the repositioning into unrelated genetic linkage groups of ohnologs. Phylogenetic analysis confirmed that all these translocated genes are true IRF9 orthologs, and not more distantly related members of the IRF family (Fig. 5). Synteny of some vertebrate genes varies among different species and some examples of such variability were described in detail. For example, NK receptor and ligand pair was found in different syntenic positions in mammals, in chicken, and in zebra finch [43]. However, location of a single gene in six non-canonical syntenic positions, each unique for a separate avian order and in the canonical position in seven additional orders is, to our knowledge, unprecedented finding for a vertebrate gene. While the evolutionary mechanisms of IRF3 and IRF9 gene losses and translocations are not known, we suspect that these phenomena are related. They could be the consequence of these genes being located before the loss/translocation on the smallest avian chromosomes, designated as dot chromosomes. Dot chromosomes have a very peculiar chromatin structure and suffer the highest rate of recombination [36, 44]. Mechanisms of the IRF3/IRF9 gene losses also seem different from the described loss of IRF10 by pseudogenization in many mammalian species, as we found no traces of any avian IRF3 or IRF9 pseudogenes [18].

**Fig. 11:**
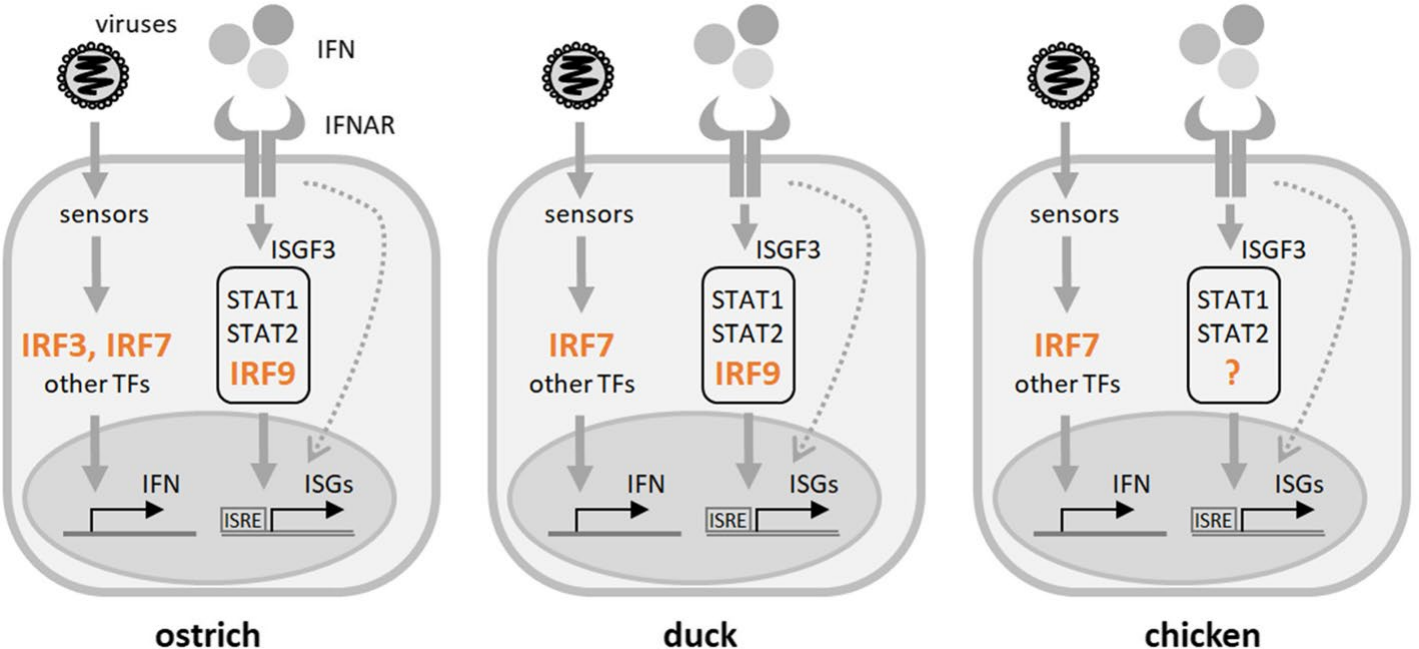
IFN induction and signaling in three avian species. A simplified schematic depiction with an emphasis on the role and presence of IRF3, IRF7, and IRF9. In the ostrich (S. camelus), all three genes are present, representing the situation in paleognath birds. The chicken (G. gallus) and other galliform birds have only IRF7. The duck (A. platyrhynchos) lacks IRF3 and serves as a representative example of a majority of birds, except for paleognaths and galliforms. The dotted arrow shows various ISGF3-independent modes of ISG induction. TF: transcription factors; IFNAR: type I IFN receptor; for other abbreviations see text

IRF genes are a typical example of a gene family that expanded due to two whole genome duplications (2 WGD) early in vertebrate evolution. While a high retention rate of IRF paralogs after 2 WGD suggests that the paralogs diversified their functions, a certain level of redundancy in function among different paralogs could still be expected. Accordingly, IRF3 function in the IFN induction pathways largely overlaps with IRF7 [45–47]. Therefore, it has been suggested that in the chicken, the role of IRF3/7 was substituted solely by IRF7 [19, 48]. As both IRF3 and IRF7 are present in paleognath birds, it will be interesting to dissect their roles experimentally in the future (Fig. 11). For example, is ostrich IRF3 expressed constitutively, and is IRF7 as IFN-inducible as it is in mammals?

IRF9 has a unique role in the ISGF3 complex, with no known substitutes in vertebrates. Therefore, its absence in the chicken and other galliform birds is quite intriguing (Fig. 11). As mentioned above, it remains possible that an extremely diverged/difficult sequence of chicken IRF9 translocated into a noncanonical syntenic position exists. In our opinion, it is much more probable that another member of the chicken IRF family may be involved in the ISGF3 complex formation. Alternatively, chicken IFN signaling might involve alternatives to classical ISGF3. Many IFN signal transduction modes independent of classical ISGF3 complex formation have been described [49, 50].

To elucidate if the avian IRF9 still retains functions in the IFN system described for this gene in mammals, we performed a series of experiments. For this work, we selected duck IRF9, because the order Anseriformes that includes duck species represents the closest relationship to galliform birds. Knockout experiments in duck embryonal fibroblasts showed that, at least in this cell type, IRF9 is essential for IFN-mediated induction of ISGs. Interestingly, residual induction of ISGs is occurring even in knockout cells. This contrasts with mammalian IRF9 knockouts, in which ISGF3-dependent ISG expression was mostly negligible [51]. For now, we can only hypothesize that the IRF9-independent induction suggests the existence of ISGF3 alternatives, possibly relevant also for the situation in IRF9-deficient chicken cells. As expected, a canonical ISRE sequence is required, together with intact IRF9, for full induction of ISGs in duck cells (Fig. 9). We suppose that duck ISGF3 binds to ISRE through IRF9-DNA interactions, similarly to mammalian ISGF3 [52, 53]. Finally, we proved the importance of duck IRF9 in a broader context than the ISG induction pathway. For this, we used the protective effect of IFN on VSV cytopathicity, a classical assay used to titer IFN activity (Fig. 10). Intact IRF9 was shown to be essential for IFN-induced antiviral responses in duck cells. This is in agreement with results obtained using mouse IRF9 knockouts [54].

## Conclusions

In summary, we have identified two previously uncharacterized members of the avian IRF family with key roles in the IFN signaling pathways. Further research will show the details of their function and the adaptations present in avian species lacking specific IRF orthologs.

## Methods

### Computational analysis of IRF genes in avian species

Sequences in the NCBI database (whole genome shotgun (WGS) genomic assemblies, nonredundant (nr) database, SRA sequences) were searched with various IRF sequences using BLAST [55]. The sequences obtained were downloaded and assembled manually, either with CLC genomics workbench 21.0.5 (Qiagen) or with Lasergene 10.0.0 (DNASTAR, Madison, WI, USA). Partial CDSs from avian genomic contigs were, if possible, completed using SRA NCBI data.

### Cell lines and culture conditions

The HEK293 T cell line was cultured in a 5% CO_2_ atmosphere at 37 °C in medium containing Dulbecco’s modified Eagle’s medium (DMEM, Sigma-Aldrich), supplemented with a 7% fetal calf serum (Gibco) and 100 μg/ml penicillin and streptomycin. Duck fibroblast cell lines were cultured in a 5% CO_2_ atmosphere at 37 °C in a medium containing a mixture of 1:1 (DMEM)/F-12 (Sigma-Aldrich), supplemented with 4% calf serum (Gibco), 4% fetal calf serum (Gibco), 1% chicken serum (Gibco), and 100 μg/ml penicillin and streptomycin.

Immortalized duck embryo fibroblasts (DEF) were prepared by standard procedure from 14 th-day-old embryos of inbred Khaki Campbell ducks [56]. For propagation of the cells, a DMEM/F12 (Gibco) medium supplemented with 5% of calf serum, 5% of fetal calf serum, 1% of chicken serum, 1% of vitamins (Sigma), 1% non-essential amino acids (Sigma), 1% folic acid (Sigma), 1% Glutamax 100 (Gibco), and 0.5% dimethyl sulfoxide was used. Secondary DEF were transfected with a pRNS1 plasmid [57] containing simian virus 40 (SV-40) DNA sequences, by a Lipofectamine 2000 reagent (Invitrogen), according to the manufacturer’s instructions. Two days later, the transfected cells were treated with a fresh medium containing G418 selection antibiotics (Sigma). A week later, the first colonies of neomycin-resistant cells appeared in the transfected culture. Starting the 14 th day after transfection, cells were regularly passaged twice a week up to passage 30. Then followed a period of slowed growth, in which the cells became highly senescent, granulated, and vacuolated, with a cell detritus in the culture medium. In this period, the cells had to be transferred at high density, but since passage 36, cells started to proliferate again and were regularly passaged three times a week. At passage 98, a stock of cells fully established for growth in vitro was frozen. As the cells exhibited very heterogeneous morphology, two rounds of cloning were performed with the aim of getting a cell population resembling secondary duck fibroblasts. In this way, a clone D7 was obtained that was further used in knockout experiments.

### Generation of the duck IRF9 knockout cell line

To create an IRF9 knockout duck cell line, we used the standard CRISPR/Cas9 technique, as described before on avian cells [58]. Duck cells (above) were seeded at a density of 8 × 10^5^ on 60 mm wells and grown to approximately 80% confluence. The gRNA-encoding sequences were prepared as sense and antisense oligonucleotides (5′-CACCGGCGGTACACCTTGTAGGGG, 5′-AAACCCCCTACAAGGTGTACCGCC), specific to the sequence at the end of duck IRF9 exon 2, mixed, phosphorylated, denatured, annealed, and ligated into the pX458 plasmid [59]. The resulting plasmid was transfected using Lipofectamine 3000 (Invitrogen). Two days after transfection, single-cell sorting into 96-well plates using the Influx cell sorter (Becton, Dickinson) was done to create GFP-positive clonal cell lines. Viable clones were expanded to a sufficient size for standard phenol–chloroform protocol DNA isolation. These clones were genotyped for successful homozygous Cas9 cleavage in the targeted region using PCR (primers 5′-GGCGTGGGCGGAGTACAAAGG and 5′-GGCTCGGCTCCGCCTCC); PCR products were verified by Sanger sequencing. For further experiments, we chose a clone with a large homozygous 141 nucleotide deletion over a splice site that deletes 56 nucleotides in the exon region. As a negative control, we chose a clone where no mutation occurred.

### Cloning and expression of duck IFN-$\alpha$

Duck IFN-$\alpha$ CDS (accession number X84764.1), an intronless gene, was amplified from genomic DNA with primers containing restriction sites *BamH*I and *EcoR*I. The digested PCR product was then cloned into a pGEM-T Easy plasmid (Promega) and verified by Sanger sequencing. To generate the final expression construct, the insert was re-cloned into a pcDNA-3.1 plasmid (GenScript Biotech Corporation). HEK293 T cells were seeded into 10-cm plates overnight to almost full confluence. They were then transfected using standard Lipofectamine 3000 protocol (Invitrogen), with a pcDNA 3.1 plasmid containing duck IFN-$\alpha$, or using the same plasmid with a GFP insert instead as a negative control. Twenty-four and forty-eight hours post transfection, medium was collected and pooled, resulting in one stock of supernatant containing duck IFN, and one stock of control supernatant. The supernatants were cleared by centrifugation and filtering (0.45 μm), and stored at − 20 °C.

### Analyzing the role of duck IRF9 in expression of ISGs

Duck WT and IRF9 KO cells were seeded at a density of 5 × 10^5^ cells per well of 6-well plates, and grown to approximately 80% confluence. Cells were treated as follows: KO cells were transfected using standard Lipofectamine 3000 protocol (Invitrogen™), either with a pcDNA 3.1 plasmid containing duck IRF9 fused with a FLAG tag on the N-terminus (GeneScript) or with a control pcDNA 3.1 plasmid containing GFP, or remained without transfection. Approximately 18 h post transfection, either supernatant containing duck IFN was added in a final dilution of 1:1000 (v/v) to both WT and KO cells, or the cells remained untreated. Six hours after IFN treatment, cells were collected for RNA isolation. The experiment was replicated twice independently; each experiment was done in three biological replicates for each experimental condition. Ectopic expression of tagged dIRF9 was verified by western blot using the mouse anti-FLAG antibody (Sigma).

### RNA isolation, reverse transcription, and quantitative PCR

Total RNA was isolated from cultured cells using RNAzol RT (Molecular Research Center), according to the manufacturer’s protocol. Reverse transcription was then performed with 0.5–1 μg of RNA, using the ProtoScript II First Strand cDNA Synthesis Kit (NEB) and 3′-RACE CDS primer (Clontech). Next, quantitative PCR was done using MESA GREEN qPCR MasterMix Plus (Eurogentec). Primers targeting duck IFIT5 (5′-AAGCTACCTTCAAACGGGTA and 5′-TCCTCCTTCAGCAAAGTCCA), Mx (5′-TCATGACTTCGGCGACAAC and 5′-AACTCGGCCACTGAGGTAAT), and GAPDH (glyceraldehyde-3-phosphate dehydrogenase; 5′-TGTCTCCTGCGACTTCA and 5′-TCCTTGGATGCCATGTGGAC) were used for expression analysis. Specificity of PCR products was verified by melting curve analysis. Each sample was analyzed in technical triplicates.

### Luciferase assays

The duck tetherin promoter region (accession number VSDN01000029.1; range: 1,112,681–1,112,965) was amplified from genomic DNA. The PCR product was then digested with primer-derived *Kpn*I and *Xho*I restriction enzymes, and inserted into the *Kpn*I/*Xho*I-digested pNL1.2-NLucP vector (Promega). The substitution G > A into the ISRE consensus sequence was introduced by In-Fusion cloning (TaKaRa). The promoter region was amplified in two fragments. The fragments were then cloned together into the pNL1.2 NLucP plasmid using the In-Fusion Cloning Kit, according to the manufacturer’s protocol. Duck WT and IRF9 KO cells were seeded at a density of 5 × 10^5^ per well into 6-well plates, and grown to approximately 80% confluence. The cells were then transfected with a plasmid containing the NanoLuc luciferase reporter gene and a promoter sequence either with consensus ISRE (GAAACGAAACT) or mutant ISRE (GAAACAAAACT). Twenty-four hours post transfection, cells were stimulated with dIFN supernatant with a final dilution of 1:1000 (v/v) for another 24 h. For luciferase signal measurement, a Nano-Glo® Luciferase Assay System (Promega) mix of lysis buffer and substrate was added to 1 × 10^5^ cells. Lysate was then transferred to white plates for a luminescence readout. The experiment was performed in biological triplicates and each replicate was measured in technical triplicates.

### Cytopathic effect inhibition assay

The cytopathic inhibition effect of duck WT and IRF9 KO cells following viral infection was tested according to Pestka et al. [41] with some modifications. The cells were seeded at a density of 1 × 10^4^ cells/100 µl into 96-well plates, and grown in the presence of the following additives: medium with supernatant from R848 (InvioGen) stimulated duck spleen cells as a positive control (diluted to 1:20 v/v), medium with supernatant containing duck IFN (see above, diluted to 1:2000 v/v), and medium with control supernatant as a negative control. After 24 h, cells were infected with Vesicular Stomatitis Virus (a gift from Mathias Büttner, Ludwig Maximilian University) with a final dilution of 1:2000, and incubated for 24 h until a clear cytopathic effect was seen in the control cells with control medium only. All media were removed from the cells and replaced by fresh medium containing neutral red at a concentration of 0.01%. After 2 h, cell culture supernatants were removed and cells were gently washed, dried, and 50 µl of a 3 M guanidine hydrochloride solution was added to solve the pinocytosed neutral red. OD was measured using an ELISA reader at a wavelength of 540 nm. Three independent assays were performed; the results represent the mean of the three assays.

### Statistics

For the qPCR and luciferase assay results, we used a nested *t*-test, and for the cytopathic effect inhibition assay, we used Welch’s *t*-test—both implemented in GraphPad Prism.

## Supplementary Information

Additional file 1. Table S1. Synteny analysis of two groups of IRF genes. Table S2. Assembled/predicted avian IRF sequences. Table S3. Vertebrate IRF sequences used for alignments and phylogenetic trees shown in figures and tables. Table S4. Search for IRF3 and IRF9 genes in genomes of Neognathae. Table S5. IRF7 sequences of neognath birds that are erroneously annotated as IRF3 in Genbank. Table S6. Vertebrate genomes used for synteny analyses

Additional file 2. Fig. S1. Percentages of amino acids with GC-rich codonsin IRF protein sequences correlate with GC contents of IRF coding sequences. Fig. S2. CLUSTAL 2.1 multiple sequence alignment of avian IRF3 proteins. Fig. S3. IRF7 sequences of neognath birds that are erroneously annotated as IRF3 in Genbankcluster with vertebrate IRF7 sequences rather than with IRF3. Fig. S4. Supplementary information for Fig. 5. Avian IRF9 protein sequences form a single clade in the phylogenetic tree. Fig. S5. Pairwise alignment of IRF9 coding sequences of endogenous duck IRF9 with in vitro synthesized construct. Fig. S6. Original gel and blot images

## Data Availability

All data generated or analysed during this study are included in this published article (and its supplementary information files).

## Abbreviations

IRF: Interferon regulatory factor
IFN: Interferon
ISG: Interferon-stimulated genes
IFNAR: Type I interferon receptor
IFNLR: Interferon lambda receptor
STAT: Signal transducer and activator of transcription
ISRE: Interferon-stimulated response element
ISGF3: Interferon-stimulated gene factor 3
2 WGD: Two whole genome duplications
NCBI: National Center for Biotechnology Information
SRA: Sequence read archive
CDS: Coding sequence
NJ: Neighbor-joining
DBD: DNA binding domain
IAD: IRF association domain
NLS: Nuclear localization signal
WGS: Whole genome shotgun
KO: Knockout
WT: Wild type
IFIT5: Interferon induced protein with tetratricopeptide repeats 5
Mx: Myxovirus resistance protein
dIRF9: Duck IRF9
Bst2: Bone marrow stromal antigen 2
VSV: Vesicular stomatitis virus
DEF: Duck embryo fibroblasts
SV-40: Simian virus 40
