# Supplementary material for: Avian interferon regulatory factor (IRF) family reunion: IRF3 and IRF9 found
Source: BMC Biol. 2025 Jul 1;23:180. doi: 10.1186/s12915-025-02261-4 (PMC12220609; doi:10.1186/s12915-025-02261-4)
Supplement: Supplementary file 2 — Additional file 2. Fig. S1. Percentages of amino acids with GC-rich codonsin IRF protein sequences correlate with GC contents of IRF coding sequences. Fig. S2. CLUSTAL 2.1 multiple sequence alignment of avian IRF3 proteins. Fig. S3. IRF7 sequences of neognath birds that are erroneously annotated as IRF3 in Genbankcluster with vertebrate IRF7 sequences rather than with IRF3. Fig. S4. Supplementary information for Fig. 5. Avian IRF9 protein sequences form a single clade in the phylogenetic tree. Fig. S5. Pairwise alignment of IRF9 coding sequences of endogenous duck IRF9 with in vitro synthesized construct. Fig. S6. Original gel and blot images [file 12915_2025_2261_MOESM2_ESM.pdf]

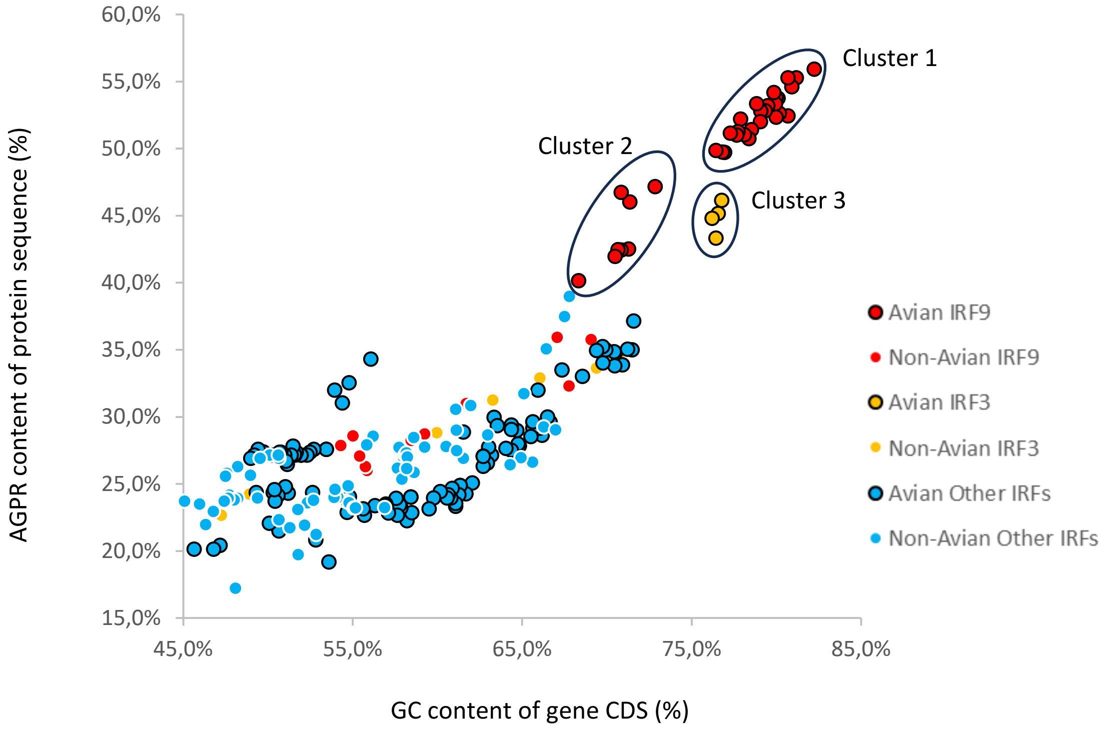

Cluster 1:

Avian IRF9 genes in original syntenic location on the dot chromosome equivalent of chicken chr 34 or avian IRF9 genes translocated to a different dot chromosome

Cluster 2:

Avian IRF9 genes translocated to either a macrochromosome or a non-dot microchromosome

Cluster 3:

Avian IRF3 genes in original syntenic position on the dot chromosome equivalent of chicken chr 31

Fig. S1 Percentages of amino acids with GC-rich codons (A, G, P, R) in IRF protein sequences correlate with GC contents of IRF coding sequences. The percentages were calculated using the sequences contained in and additional file (Additional file 1: Table S3). Data from all genes where calculation of GC and AGPR percentages are shown in the table were included. Duplicate values were eliminated. Percentages were not calculated for genes with inadequately complete sequences.

```
dnoIRF3     MGAPKPLILPWLRAQLDRGGYPGVRWLG--ATRFRVPWKHGLRHDVAPEDFQLFQDWAIASGCYRPGVDPPAPAVWKRNFRAALNRKPGLRVVEDRSGDAGDPHKVYEFLPGGPGGAGDP
scaIRF3     MGTPKPLILPWLRAQLDRGCYPGVRWLG--ATRFRVPWKHGLRHDVAPEDFQLFQDWAIASGCYRPGVDPPVPAVWKRNFRSALNRKPGLRMLEDHSGDANDPHKVYEFLA---------
amaIRF3     MGTPKPLILPWLRAQLDRGGYAGVRWVG--ATRFRVPWKHGLRHDVAPEDFQLFQDWAIASGCYRPGVDPPAPAVWKRNFRAALNRKPGLRVVEDRSGDAGDPHKVYEFLP---------
rpeIRF3     MGNPKPLILPWLRAQLDRGTYPGVRWLG--GTRFRVPWKHGLRHDVAPEDFQLFQDWAIASGCYRPGVDPPAPAVWKRNFRAALNRKPELRVVEDRSGDAGDPHKVYEFLP---------
asiIRF3     MGSQRPLLVPWLREQLDSGCYPGVCWLNAERTRFRVPWKHGLRHDAAREDFQLFQDWATVSGCYHAG-TAPVPSIWKRNFRSALNRKPEFKVVEDNSSDATDPHKVYEILP---------
ccarIRF3    MGTPKPLIVPWLRKKLDDGCYPGVSWLDQGRTQFRVPWKHGLRQDASSEDFQLFRDWAIDSGNYRPGHDAPTPSIWKRNFRSALNRKPGIQVLQDHSSDSADPHKVYEILP---------
hsaIRF3     MGTPKPRILPWLVSQLDLGQLEGVAWVNKSRTRFRIPWKHGLRQDAQQEDFGIFQAWAEATGAYVPGRDKPDLPTWKRNFRSALNRKEGLRLAEDRSKDPHDPHKIYEFVNS--------
mmuIRF3     METPKPRILPWLVSQLDLGQLEGVAWLDESRTRFRIPWKHGLRQDAQMADFGIFQAWAEASGAYTPGKDKPDVSTWKRNFRSALNRKEVLRLAADNSKDPYDPHKVYEFVTP--------
cfaIRF3     MGTPKPRILPWLVSQLDLGQLEGVAWLDERRTRFRIPWKHGLRQDAQQEDFGIFQAWAEVSGAYTPGKDKPDLPTWKRNFRSALNRKEELRVAEDRSKDPHDPHKVYEFVIS--------
            *   :* ::***  :** *   ** *:.   *:**:*******:*.   ** :*: **  :* * .*   *  . ******:*****  :::  *.* *. ****:**::

dnoIRF3     PEVYEFLPGALGGASGAGDPPETYEFLPGVPGGASGAGDPPEVYEFLPGVPGG----AGQPPRAGDAGPAEPPEASGGDLELLE----PELDCGQDWDLDEVMNMLVLDSPEEDVAISLL
scaIRF3     ------------------------------------------------EVPGG----PGQPPGAGAAGPEEPPEXAGGDLELLE----PELDCGQDWDLDEVMNMLVLSSPEEDXAISLL
amaIRF3     ------------------------------------------------QAPGGGDGPPQAPPEEGGAGPEEP---PGGALELLEPELELELDCDQDWDLDEVMNMLVLSSPDENAAISLL
rpeIRF3     ------------------------------------------------EAPG-------QPPGAGGAGPEMPPEVPGGDLEDLE-LLEPELDCGQDWDLDEVMNMLVLSSPDENAAISLL
asiIRF3     -----------------------------------------------VGVQAG----ADEAPLVAGRADTLD-----GPLEQLL-GEG----CSPDSDLEQYVHLMTLCSPEEDISSPDL
ccarIRF3    -----------------------------------------------NGARAG----ADEAPSAAVGGTEAD-----ASSQLLD-RSGGGLSCSQDDDLDDILNALALSSPEEDAPCSAL
hsaIRF3     ------------------------------------------------GVGDFSQ--PDTSPDTNGGGSTSD---------------------TQEDILDELLGNMVLA-PLPDPGPPSL
mmuIRF3     ------------------------------------------------GARDFVH--LGASPDTNGKSSLPH---------------------SQEN-LPKLFDGLILG-PLKDEGSSDL
cfaIRF3     ------------------------------------------------GAGNLPE--LDTFPDTNGRYSTSD---------------------TQEDTLEELLGDMVLT-PFPDEGPSSL
                                                            .               *                        :  * . .  : *  *  :   . *

dnoIRF3     APAPDDAPDPPVLPPDPPYCPPEDAMGLGPPPQP-GCPLP-DLLIGADELRTDLEVRVYYRGRAVLQTTVAAP-GQLLLVAPGTPGTPV---APGTVALPEPA--VADGRQARYTRRVLR
scaIRF3     APAPDAAPDPSLLLPDPPYCPPEDAVGLGPPLQP-SSPLP-DLLIGADELRTDLEVRVYYRGRPVLQAAVAAP-GELRLVPP--PGPPG---AAGTVALPEPA--VADGRQARYTRRVLQ
amaIRF3     APAPDAAPDPPALPPDPPYCPPEDAMGPAPPLQP-SSPLP-ELLIGADELRTDLEVRVFYRGRAVLETAVAAAAGGLRLVAAAAAGGAAGGAAPGTVALPEPA--VADRRQAHYTRRVLR
rpeIRF3     GPAPPAAPGPPALLPDPPYCPPEEAVGPPPALQP-SSPLP-ELLIGADELRTDLEVRVYYRGRPVLQATVAAAPEGLRLAAPGPAASAGVGAA---VPLPEPE--VPDGRQARYTRRVLQ
asiIRF3     SL---FGPGEAVGDAAVP-VLS-GAEVLPPLVQPLSTPNPGGLLLGSNELVTDFDVNVYYRGHRVLHTLVSSS-QGLRLVFPQSSPSPAP--ELTDVVLPDPES-LPDRVQANYTGRLLR
ccarIRF3    AP---GAP--YVGDAATGGALSPGFTDFPPLVQP--APSALEQLLGSNVLMTDFEVRVYYRGHLVLQTLVPNP-QGFRLVSTSPGPSPCP--ELVDVELPGPGM-LPDRVQASYTARLLQ
hsaIRF3     AV---------APEPCPQPLRSPSLDNPTPFPNLGPSENPLKRLLVP-GEEWEFEVTAFYRGRQVFQQTISCP-EGLRLVGSEVGDRTLP---GWPVTLPDPGMSLTDRGVMSYVRHVLS
mmuIRF3     AI---------VSDPS-QQLPSPNVNN---FLNPAPQENPLKQLLA--EEQWEFEVTAFYRGRQVFQQTLFCP-GGLRLVGSTA-DMTLP---WQPVTLPDPEGFLTDKLVKEYVGQVLK
cfaIRF3     VV---------VPEQTPPLLLSPTIDLPAPCPNSEPPENPLKRLLVP-DEEWEFEVTAFYRGRQVFQQTVSCP-RGLRLVAAAGGDTMLP---GQPIILPDPGVLVTDKTVMGYVRRVLS
                                .            :       .    *:        :::* .:***: *:.   :   .    : *. .                : ** *    :.*     *. ::*

dnoIRF3     GLGAG--------------VGLRTRGPGLEGARLGCCRVFWGRGGTPG---PGPPRGALPKGTYGPLYGLRDFVRELIAFMEGGGESPQYELWLCLGEPWPEPGHSWTRKLIMVQVVPVA
scaIRF3     GLGAG--------------VGLRARGPALEGTRLGRCRVFWGRGGGPG---PGAPGGALPKGTYGPLYGLRDFVRELIAFLEGRGGSPHYELWLCLGEPWPEPSHSWTRKLVMVQVVPVA
amaIRF3     GLGGGGGGDGDGGDDAGGGVTLRPRGPALEGARLGRCRVFWGRGGGPG--GPETPGGALPKGAYEPLYGLRDFVRELIAFMEGRGGSPQYELWLCLGEPWPQPSHTWTRKLVMVQVVPVA
rpeIRF3     GLGAG--------------VALRARGPELEGARLGRCRVFWGRGGGGNP-GPGAPGGALPKGTYGPLYGLRDFVRELIAFMEGRGGHPXYELWLCLGESWPEPGHHWTRKLVMVQVVPVA
asiIRF3     GLGPG--------------VVLRAKGRILGGCRLGRCHAFWGLTATPA---PGALGGEIPKESFTPLYSLPEFIQDLIGFMEGQRRSPDYELWLCLGEKWPDTERPWNRKLIMVQVVLVA
ccarIRF3    GLGAG--------------VLVRVEGPALCATRLGRLHAYWGHTETPE---PGAEGGELSKEGYTRLYDMSHFVRELICFIEGQGSSPTYELWLCLGEAWPDTGRSWKKKLIMVQVVPTV
hsaIRF3     CLGGG--------------LALWRAGQWLWAQRLGHCHTYWAVSEELLPNSGHGPDGEVPKDKEGGVFDLGPFIVDLITFTEGSGRSPRYALWFCVGESWPQ-DQPWTKRLVMVKVVPTC
mmuIRF3     GLGNG--------------LALWQAGQCLWAQRLGHSHAFWALGEELLPDSGRGPDGEVHKDKDGAVFDLRPFVADLIAFMEGSGHSPRYTLWFCMGEMWPQ-DQPWVKRLVMVKVVPTC
cfaIRF3     CLGGG--------------LALWRAGQQLWARRLGHCHTYWALGEELLPDSSPRPGGEVPKDEDGDLFDLRPFVSDLIAFIKGSRHSPRYTLWFCVGEPWPQ-DQPWTKKLVMLKVVPTC
             ** *              : :   *   * . ***  :.:*.              * : *    ::.:  *: :** * :*    * * **:*:** **:  : * ::*:*::** .

dnoIRF3     LRLLHELSRAQGASSLRSSELQLQLSASLG-----EGGLLGALRSWDERMDSLPSA--
scaIRF3     LRLLHELSQEQGASSLRSSELQLQLSASLG-----PGGLLGALRGWGERMDTLPXA--
amaIRF3     LRLLHELSRAQGASSLRSSELQLQLSASLG-----EGGLLGALRSWDEHMDSLPPP--
rpeIRF3     LRLLHELSRAQGASSLRSSELQLQLSASLDGADAGGGGLLGALRSWDERMDS------
asiIRF3     LQKLHELSQAGGASSLRSSELNLHISDSLG-----DGGLLGALRSWEELMDTQP----
ccarIRF3    LRTLHELGQAGGASSLRGEELDLRISDSLG-----EAGLLGALRDWEERMESQPYS--
hsaIRF3     LRALVEMARVGGASSLEN-TVDLHISNSHP-LSLTSDQYKAYLQDLVEGMDFQGPGES
mmuIRF3     LKELLEMAREGGASSLK--TVDLHISNSQP-ISLTSDQYKAYLQDLVEDMDFQATGNI
cfaIRF3     LRALLEMARLEGASSLEN-TVDLHISNSYP-LTLTSDQYKAYLQDLVEDMDFWVTGEV
            *: * *:.:  *****.   ::*::* *              . *:.  * *:
```

Fig. S2 CLUSTAL 2.1 multiple sequence alignment of avian IRF3 proteins (dno – *Dromaius novaehollandiae*, emu; sca – *Struthio camelus*, common ostrich; ama – *Apteryx mantelli*, North Island brown kiwi; rpe – *Rhea pennata*, Darwin's rhea) with IRF3 proteins from non-avian archelosaurs (ccar –

Caretta caretta, loggerhead sea turtle; asi – *Alligator sinensis*, Chinese alligator) and mammals (hsa – *Homo sapiens*, human; mmu – *Mus musculus*, house mouse; cfa – *Canis familiaris*, dog). Exonic structures of CDS are shown by alternating black and blue ink. Amino acids with codons split by introns are shown in red ink; in the case of phase 0 exon/intron boundaries, both bordering amino acids are bold. Five conserved tryptophans in DBD are indicated by a cyan highlight while two conserved serines in the C-terminus instrumental for activation of IRF3 by phosphorylation are indicated by a yellow highlight. Four-fold repetitive structure of the extension of the second exon of emu (dno) IRF3 is visualized using alternating solid and wavy underlines.

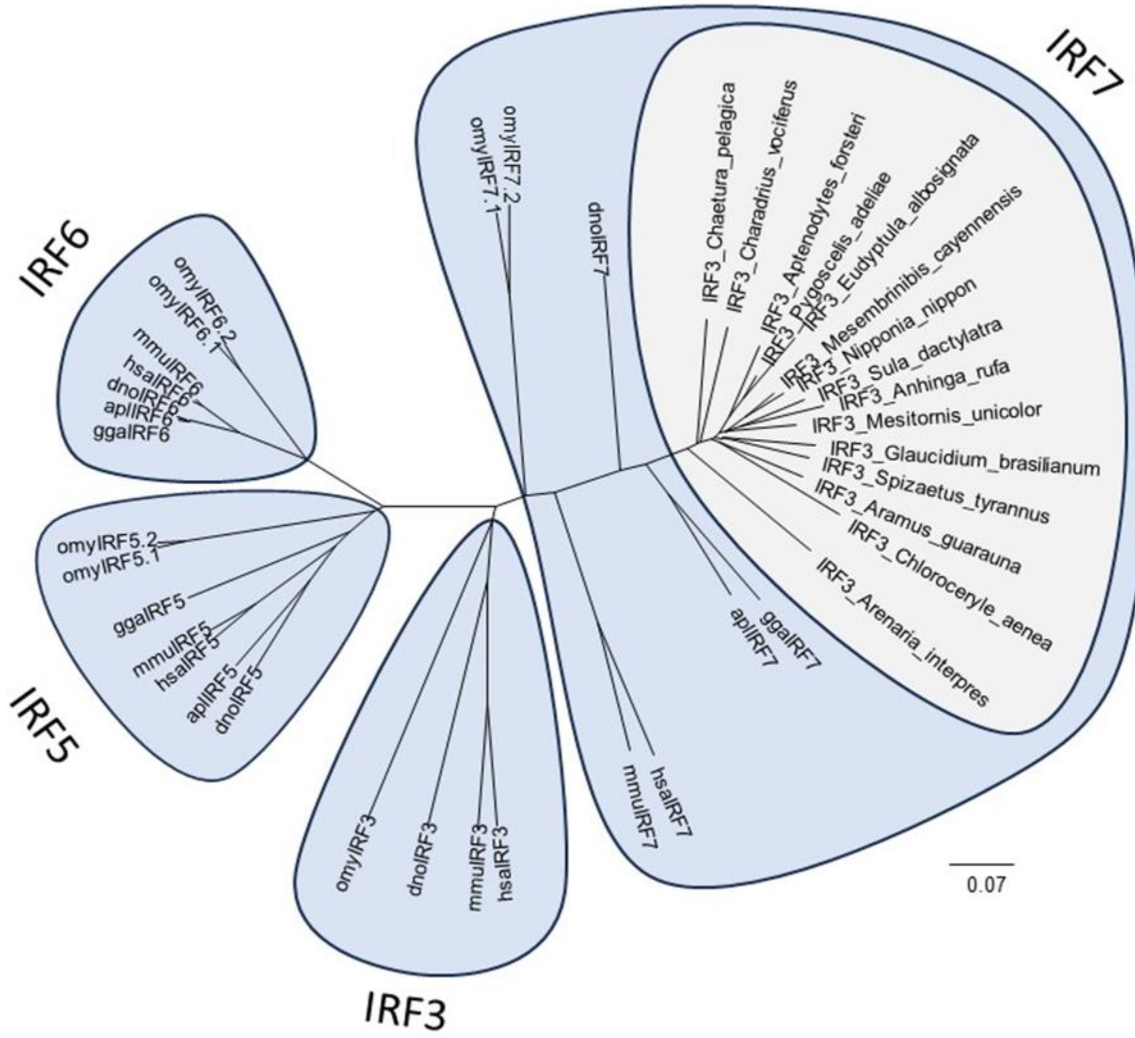

Fig. S3 IRF7 sequences of neognath birds that are erroneously annotated as IRF3 in GenBank (shown on gray background) cluster with vertebrate IRF7 sequences rather than with IRF3. NJ tree of Clustal X-aligned protein sequences. False IRF3 sequences are listed in an additional file (Additional file 1: Table S5). Control IRF sequences come from the following species: apl - Anas platyrhynchos (duck), dno - Dromaius novaehollandie (emu), gga - Gallus gallus (chicken), hsa - Homo sapiens (human), mmu - Mus musculus (mouse), omy - Oncorhynchus mykiss (rainbow trout), and are available an additional file (Additional file 1: Table S3).

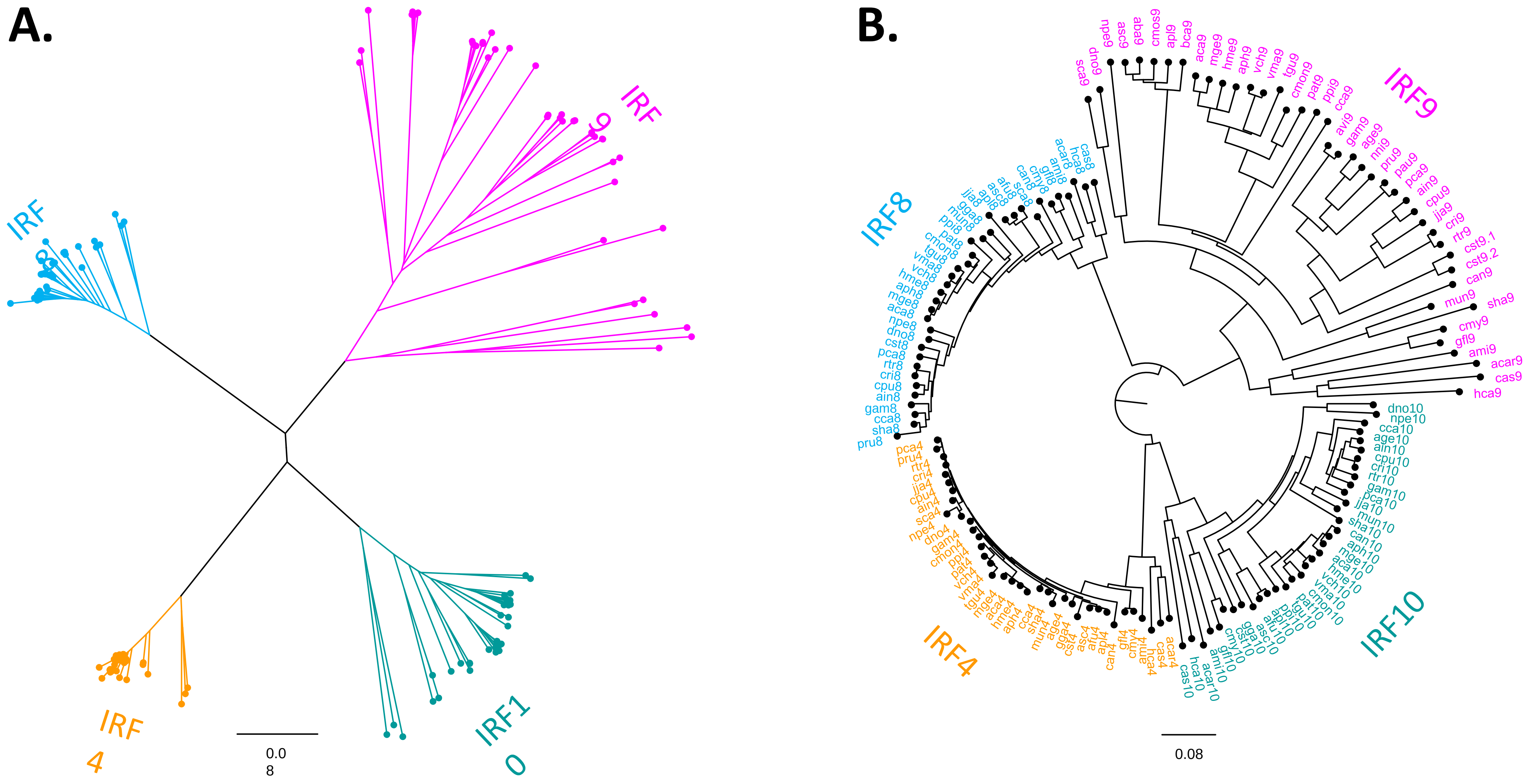

Fig. S4 Supplementary information for Fig. 5. Avian IRF9 protein sequences form a single clade in the phylogenetic tree. **A.** The tree shown in the same form of radial phylogram as is shown in Fig.5 in the main text. Each IRF family member is, however, distinctly color-coded. **B.** The same tree shown in form of circular cladogram with species labels included. For species labels look in the additional file (Additional file 1: Table S3).

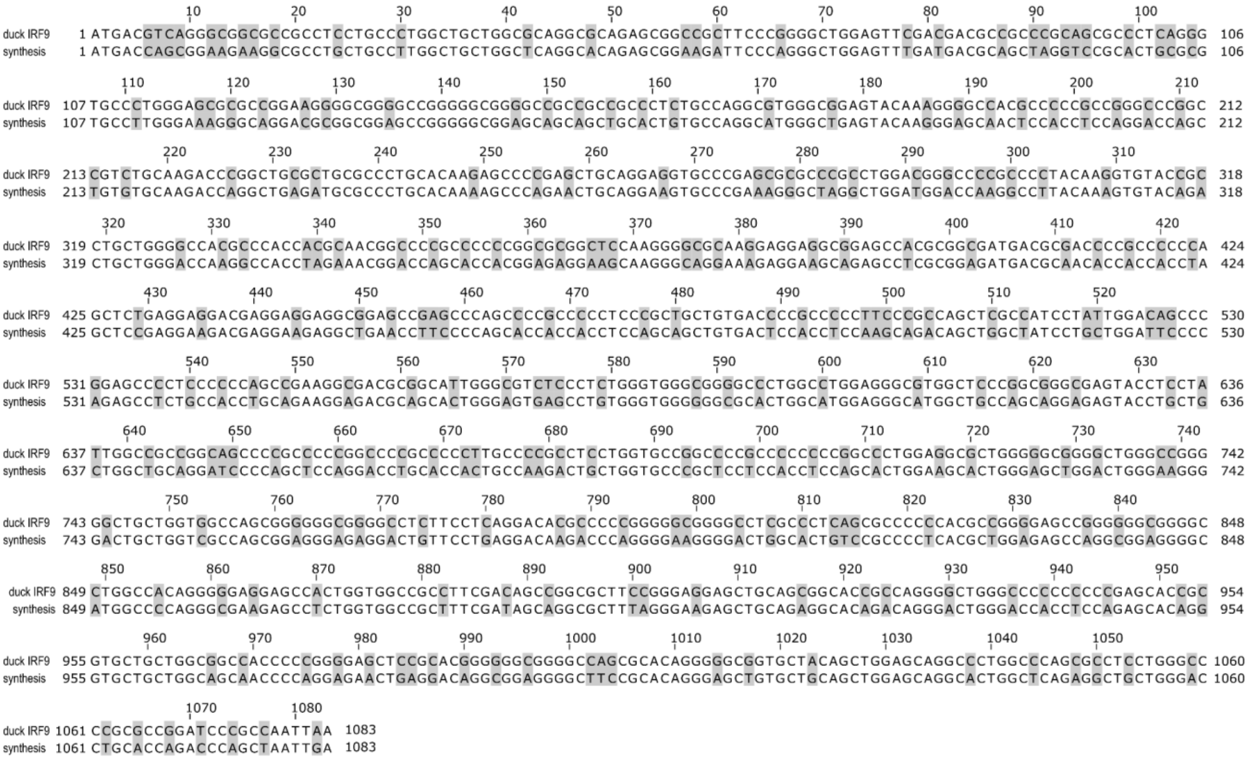

Fig. S5 Pairwise alignment of IRF9 coding sequences of endogenous duck IRF9 with in vitro synthesized construct. The predicted protein coding sequence is identical for both. In the gene synthesis construct, the codons were optimized to break GC-rich stretches. Mismatches in the nucleotide sequence are highlighted in gray color.

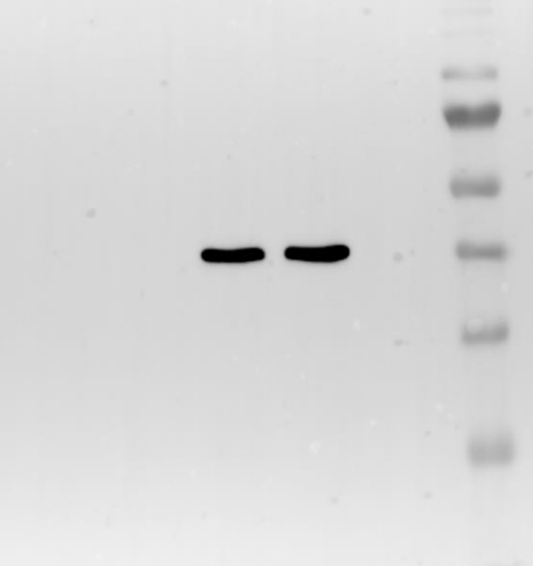

43 kDa

-	-	+	+	pcDNA3.1_IRF9
KO	KO	KO	KO	

(ad Fig. 7B)

Coomassie blue, 12% gel

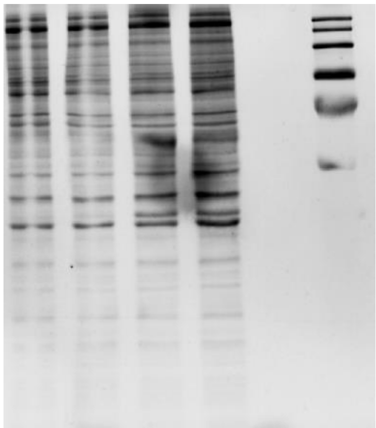

Fig. S6 Original gel and blot images. Western blot of lysate from duck KO cells transiently transfected by the dIRF9 expressing plasmid and corresponding Commassie blue gel from figure 7B are shown.
